# Dual-Gradient Impedance/Insulation Structured Polyimide Nonwoven Fabric for Multi-Band Compatible Stealth

**DOI:** 10.1007/s40820-025-01966-z

**Published:** 2026-01-04

**Authors:** Xinwei Tang, Wei Hong, Hongmiao Gao, Shuangshuang Li, Wei Li, Kaixin Lai, Mingzhen Xu, Zaiyin Hu, Yan Li, Zicheng Wang, Tianxi Liu

**Affiliations:** 1 The Key Laboratory of Synthetic and Biological Colloids, Ministry of Education, School of Chemical and Material Engineering, International Joint Research Laboratory for Nano Energy Composites, Jiangnan University, Wuxi, 214122 Jiangsu People’s Republic of China; 2 Yangtze Delta Region Institute (Huzhou), University of Electronic Science and Technology of China, Huzhou, 313001 Zhejiang People’s Republic of China; 3 Guizhou Aerospace Wujiang Electro-Mechanical Equipment Co., Ltd., No. 20-5, Dalian Road Aerospace Industrial Park, Huichuan District, Guizhou, 563100 Zunyi People’s Republic of China; 4 Jiangsu Ferrotec Semiconductor Technology Co., Ltd., Yancheng, 214000 Jiangsu People’s Republic of China

**Keywords:** Polyimide, Electroless plating, Electromagnetic interference shielding, Radar stealth, Infrared stealth

## Abstract

Designing and preparing a compatible electromagnetic interference (EMI) shielding, radar and infrared stealth material exhibits significant prospect in the military field. Hence, a novel conductive/magnetic polyimide-based nonwoven fabric (PFN_y_) is prepared by alkali treatment, Fe^3+^ ion exchange, thermal reduction, and electroless nickel (Ni) plating process. Its impedance/insulation characteristics can be easily adjusted by controlling the in situ growth of Fe_3_O_4_ and electroless nickel plating. Subsequently, a new strategy of constructing hierarchical dual-gradient impedance/insulation structure is implemented to achieve EMI shielding, radar and infrared stealth via stacking PFN_y_ with gradually decreased impedance/insulation characteristics from top to bottom. The formation of impedance matching gradient structure promotes effective introduction and dissipation of electromagnetic waves, endowing the composite with outstanding EMI shielding and radar stealth performance. Meanwhile, the construction of thermal insulation gradient structure can effectively inhibit thermal radiation from target, bringing an excellent infrared stealth performance. Importantly, the strong interfacial interactions between Fe_3_O_4_, Ni and polyimide fiber accelerate PFN_y_ to resist the stresses originated from high-temperature heat source, achieving a compatible high-temperature resistant radar/infrared stealth performance. Such excellent comprehensive properties endow it with a great potential in high-temperature military camouflage applications against enemy radar and infrared detection.

**Supplementary Information:**

The online version contains supplementary material available at 10.1007/s40820-025-01966-z.

## Highlights

- A novel conductive/magnetic polyimide nonwoven fabric is prepared by alkali treatment, Fe^3+^ ion exchange, thermal reduction, and electroless nickel plating process.
- Dual-gradient impedance/insulation structure is assembled through stacking different conductive/magnetic polyimide nonwoven fabrics with gradually decreased impedance/insulation characteristic from top to bottom.
- The strong interfacial interaction and dual-gradient impedance/insulation structure bring a compatible electromagnetic interference shielding, high-temperature resistant radar and infrared stealth performance.

**Supplementary Information:**

The online version contains supplementary material available at 10.1007/s40820-025-01966-z.

## Graphical abstract

**Figure:**
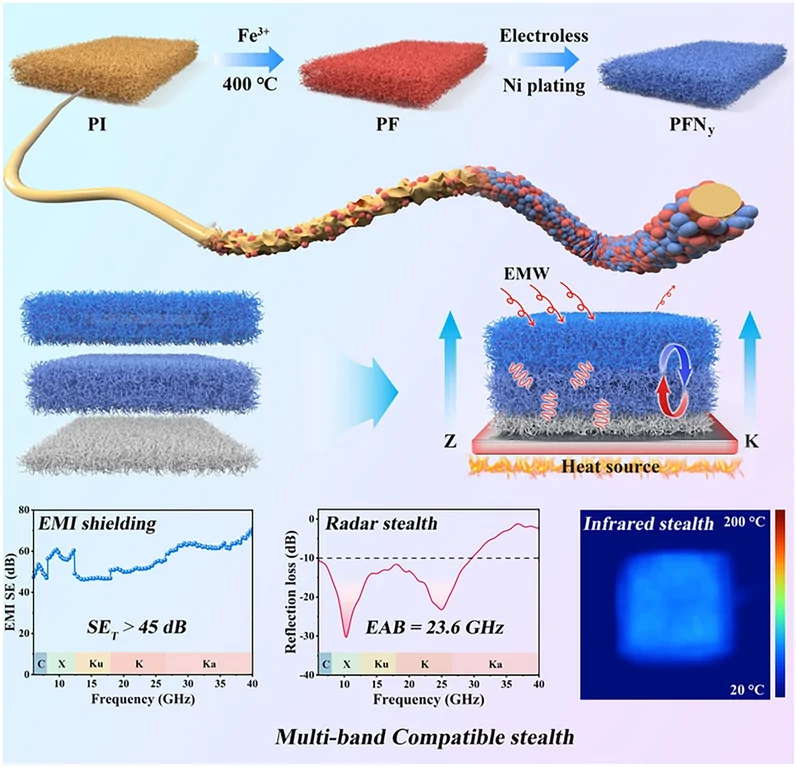

## Introduction

With the rapid development of electromagnetic detection technologies, the exposure potential of military targets is gradually increasing [1, 2]. The electronic system in military equipments generates serious electromagnetic wave (EMW) reflections and distinct infrared radiation, which are easily captured by enemy radar and infrared detectors [3–6]. Furthermore, the EMW radiated by aircraft communication modules and/or other electronic systems can be exploited by hostile electronic intelligence systems to accurately locate the aircraft [7, 8]. Therefore, designing and fabricating compatible electromagnetic interference (EMI) shielding, radar and infrared stealth materials become a significant focus in the field of military camouflage.

Typically, it is critical to realize radar stealth by efficient introducing and dissipating EMW originated from enemy. As reported by studies, the rational construction of impedance matching structure is considered as effective way to realize the above requirements [9–11]. A classic example is the cellulose nanofiber/Fe_3_O_4_ (CNF/Fe_3_O_4_) hydrogel by controlling Fe_3_O_4_ content gradients to achieves a minimum reflection loss (*RL*_min_) of − 59.5 dB and an effective absorption bandwidth (*EAB*) of 5.2 GHz at a thickness of 1.9 mm [11]. On the other hand, according to Stefan–Boltzmann law of infrared (IR) radiation: *E* = *εσΤ*^*4*^, where *E* is the IR radiation intensity of materials, *ε* is the IR-emissivity of materials, *σ* is the Stefan–Boltzmann constant, and *Τ* is the absolute surface temperature of materials [12–15]. Thus, reducing IR-emissivity (*ε*) or thermal conductivity (*λ*) is required. For example, a MXene/black phosphorus/Ni-MXene (MXene/BP/Ni-MXene) film presents a low IR-emissivity (0.1) and excellent infrared stealth performance [14]. However, lower IR-emissivity brings a high electrical conductivity of 108.7 S cm^−1^, causing severe impedance mismatch and EMW reflection (*R* ~ 0.8). Due to the large specific surface area and ultrahigh porosity, porous materials (aerogel and/or foam) exhibit a low thermal conductivity, thus enabling excellent infrared stealth performance. The aerogel fiber synthesized by Zhang et al. displays an ultrahigh porosity (99.7%), ultralight density (3 mg cm^−3^) and low thermal conductivity (25.3 mW m^−1^ k^−1^), which effectively suppresses thermal radiation (*ΔT* ≈ 30 °C at 100 °C) [15]. However, the pure polymer-based aerogel exhibits low electromagnetic dissipation capability. Therefore, electromagnetic dissipation fillers should be introduced into the matrix, achieving effective dissipation for incident EMW. For instance, Li et al. successfully develop a MXene/Polyimide aerogel through freeze-drying method [16]. As a consequence, the composite exhibits excellent microwave absorption (MA) performance (*RL*_min_ = − 49.36, *EAB* = 6.9 GHz) at a thickness of 1.92 mm. However, the thermal conductivity of composite aerogel is inevitably increased, owing to the introduction of electromagnetic dissipation fillers. Therefore, the composite must be thickened (> 10 mm) to insulate the high-temperature thermal radiation for excellent infrared stealth performance [17–19]. Unfortunately, the larger thickness hinders practical applications. Additionally, those electromagnetic dissipation fillers are introduced into matrix by in situ composite or vacuum-impregnation approach [20–24]. In situ composite method enhances the interfacial interaction between fillers and matrix, but dramatically degrade the mechanical properties of matrix due to high fillers contents. Vacuum impregnation approach maintains the mechanical properties, but the weak interfacial interaction fails at high temperatures. Notably, most MA materials typically exhibit low EMI shielding performance. Thus, how to construct robust interfacial interaction composites with compatible EMI shielding, radar and infrared stealth performance becomes a critical challenge.

Hence, a novel conductive/magnetic polyimide nonwoven fabric (PFN_y_) is prepared by alkali treatment, Fe^3+^ ion exchange, thermal reduction, and electroless nickel plating process. The impedance/insulation characteristics of PFN_y_ can be easily adjusted by controlling the in situ growth of Fe_3_O_4_ and electroless nickel plating process. Subsequently, a new strategy of constructing hierarchical dual-gradient impedance/insulation structure is employed to achieve EMI shielding, radar and infrared stealth via stacking PFN_y_ with gradually decreased impedance/insulation characteristic from top to bottom. The formation of impedance matching structure promotes the effective introduction and dissipation of EMW, endowing the composite with an outstanding EMI shielding (SE_T_ > 45 dB in 6–40 GHz) and radar stealth (*RL*_min_ = -23.87 dB, *EAB* > 22.1 GHz) performance. Meanwhile, the construction of thermal insulation gradient structure and three-dimensional (3D) fluffy network structure of nonwoven fabric can endows it with a low thermal conductivity (0.0659 W m^−1^ K^−1^), bringing an excellent infrared stealth performance. The construction of dual-gradient structure balances the paradox between the electromagnetic dissipation capacity and thermal conductivity. More importantly, the formation of strong interfacial interactions between Fe_3_O_4_, Ni and PI fiber accelerates PFN_y_ to resist stress originated from high-temperature heat source, achieving a compatible high-temperature resistant radar/infrared stealth performance. The dual-gradient strategy effectively integrates EMI shielding, radar, and infrared stealth performance, offering promising applications in high-temperature military camouflage.

## Experimental Section

In a typical experiment, pure PI nonwoven fabrics with different thicknesses are prepared by electrostatic spinning machine (ET-2535H, Beijing Ucalery Co.,Ltd., China), as reported by previous work [25]. And a conductive/magnetic PI nonwoven fabric (PFN_y_) is prepared by alkali treatment, Fe^3+^ ion exchange, thermal reduction, and electroless Ni plating process. Subsequently, the hierarchical dual-gradient impedance/insulation structure (PFN_x–y–z_) is assembled through stacking PFN_y_ with gradually decreased impedance/insulation characteristic from top to bottom. The adopted materials, experimental steps and characterization are provided in Supporting Information.

## Results and Discussion

### Structure and Morphology of Nonwoven Fabric

**Fig. 1:**
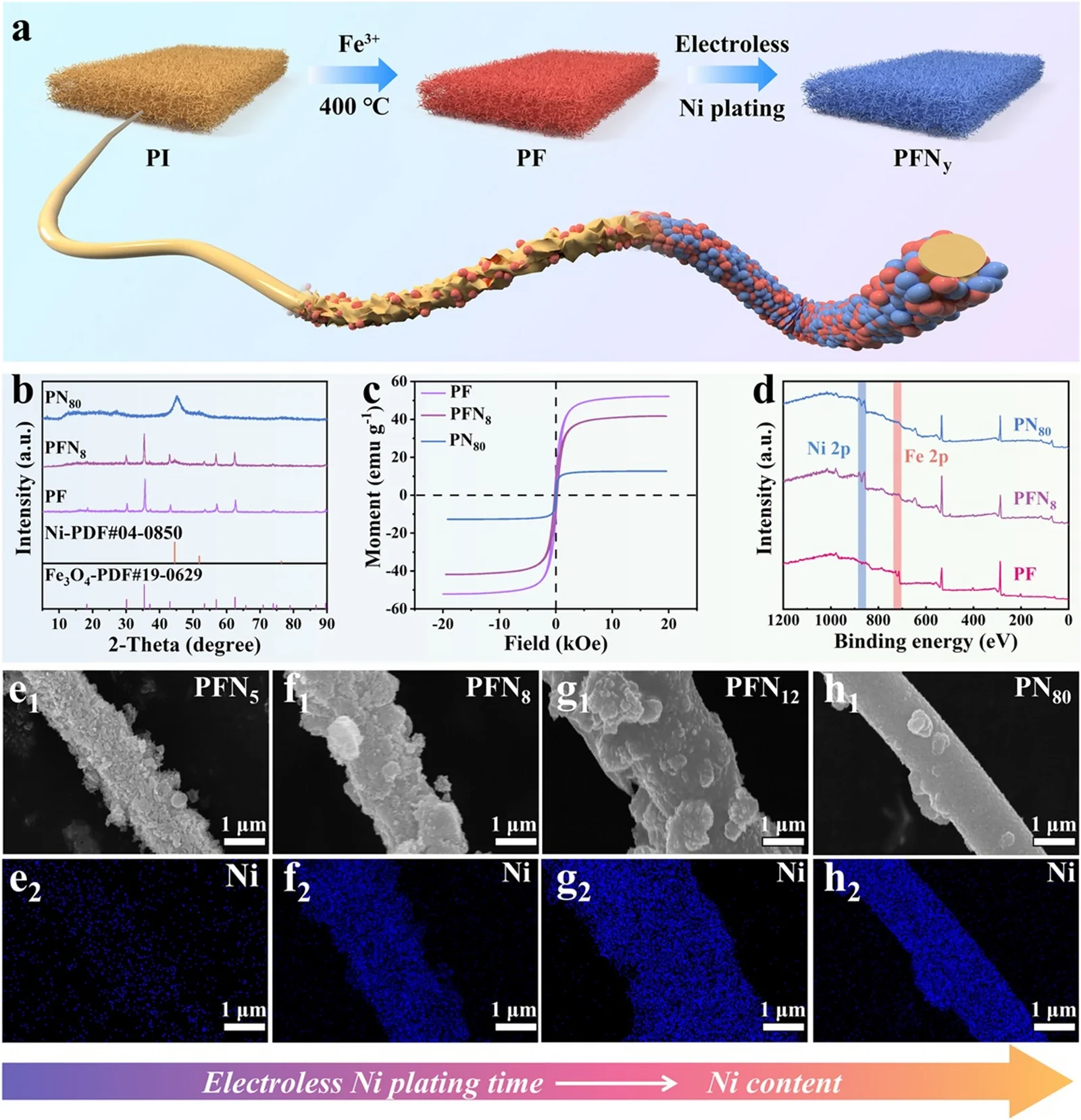
**a** Schematic illustration of the fabrication process of conductive/magnetic polyimide nonwoven fabric (PFN_y_). **b** XRD patterns, **c** Hysteresis loops, and **d** XPS spectra of PF, PFN_8_ and PN_80_. SEM and EDS mapping images of **e** PFN_5_, **f** PFN_8_, **g** PFN_12_ and **h** PN_80_

A novel conductive/magnetic PI nonwoven fabric (PFN_y_) is prepared by alkali treatment, Fe^3+^ ion exchange, thermal reduction, and electroless nickel plating process as demonstrated in Fig. 1a. Firstly, pure PI nonwoven fabrics with different thicknesses are prepared as reported [25]. As shown in Fig. S1, a smooth surface fiber is observed with a diameter of ~ 1 μm. Meanwhile, the chemical structure of PI nonwoven fabric is characterized by FT-IR and exhibited in Fig. S2. A series of characteristic peaks can be observed, which corresponds to the C=O stretching vibrations (1779 and 1716 cm^−1^), C–N stretching vibration (1317 cm^−1^), and deformation vibration (723 cm^−1^) of imide ring, respectively. It indicates that polyimide is successfully prepared by thermal imidization process. Subsequently, nonwoven fabric is treated by NaOH to hydrolyze surface imide rings into carboxyl groups (–COOH) on PI fibers [26, 27]. As shown in Fig. S3, a relatively rough surface can be obtained on PI fiber after NaOH treatment. These groups provide active sites for adsorption of Fe^3+^ during ion exchange process. Subsequently, magnetic nanoparticles can be generated by thermal reduction treatment. The in situ growth can provide a strong interfacial interaction between magnetic nanoparticle and PI fiber. As exhibited in Fig. S4, a compact and rough nanoparticle appears on the surface of PI fibers after thermal treatment at H_2_/Ar atmosphere. The obtained composite is labeled as PF. Energy-dispersive X-ray spectroscopy (EDS) analysis is employed to characterize the element changes on the surface of PI fiber. It can be obviously seen that Fe and O elements are uniformly distributed on the surface of PI fiber (Fig. S5). It indicates that Fe_3_O_4_ is uniformly in situ grown on the surface of PI fibers. Meanwhile, X-ray diffraction spectra (XRD) are carried out. As demonstrated in Fig. 1b, a series of visible characteristic diffraction peaks arises in 30°, 35°, 43°, 53°, 56°, and 62°, which correspond to the indexed planes of Fe_3_O_4_. Furthermore, the vibrating-sample magnetometer (VSM) is performed to characterize the magnetic properties of PF. As displayed in Fig. 1c, PF exhibits a significant saturation magnetization (Ms) of 52.16 emu g^−1^. In addition, X-ray photoelectron spectroscopy (XPS) is characterized to explore the surface chemical change of nonwoven fabric. As shown in Fig. 1d, the characteristic peak of Fe 2*p* can be observed in XPS spectrum of PF. The high resolution XPS spectrum of Fe 2*p* for PF can be further resolved into four characteristic peaks (Fig. S6), including 710.01, 713.60, 723.34, and 727.78 eV [28, 29]. Meanwhile, two satellite peaks appear at 717.81 and 732.76 eV. It indicates that the valence state of iron in PF is Fe^3+^ and Fe^2+^. The area ratio of spin–orbit splitting peaks is close to 2:1. It once again confirms that Fe_3_O_4_ successfully in situ grows on the surface of PI fibers. In addition, the thermal stability of PF is characteristic by TGA. As shown in Fig. S7, for PI and PF, no obvious thermal decomposition occurs before 400 °C. It indicates that PI and PF possess excellent thermal stability. Meanwhile, an increased residual yield of ~ 56.0% for PF can be obtained compared to that (~ 49.5%) of PI at 1000 °C. It can be ascribed to a fact that the introduction of Fe_3_O_4_ with higher thermal stability in PI nonwoven fabric. Moreover, the quantitative result further confirm that Fe_3_O_4_ is successfully in situ grown on the surface of PI fiber.

Secondly, to further enhance electromagnetic dissipation capacity, electroless Ni plating is employed to enhance the permittivity and permeability of PF. In this system, a strong adhesion PDA roughened coating forms on the surface of PF by oxidative self-polymerization of dopamine (DA). Very importantly, the catechol and amine groups in PDA brings more active ligand/reduction sites for PF, providing a “chemical” anchor for subsequent electroless Ni plating [30, 31]. It effectively promotes the in situ immobilization/reduction of Pt^4+^ to Pt nanoparticles. The Pt nanoparticle serves as catalytic center to accelerate nickel atom deposition during electroless Ni plating process [25]. As shown in Figs. 1e_1_-g_1_ and S8a-d, the surface of PF fiber becomes smoother as the electroless plating time increases from 5 to 12 min. Meantime, the corresponding diameter of fibers exhibits a tendency to increase with the increasing electroless plating time from 5 to 12 min. Correspondingly, element distribution of Ni shows a significant tendency to increase, while that of Fe decreases (Figs. 1e_2_-g_2_ and S9a-c). It is worth noting that Fe and Ni elements are uniformly distributed on the surface of PI fibers. It can be concluded that Fe_3_O_4_ and Ni exhibit a uniform state on the surface of PI fibers. It adequately validates the uniform distribution of Fe_3_O_4_ and Ni. It ensures the precise replication of fiber architecture. In addition, as the electroless Ni plating time increases, the residual yield of PFN_5_, PFN_8_, and PFN_12_ exhibits a tendency to increase from 61.1% to 68.5%, and then to 70.2%, respectively (Fig. S7). These quantitative results indicate that more Ni nanoparticles are loaded on PF fibers, as the electroless Ni plating time is extend from 5 to 12 min. The Ni content can be effectively controlled by extending electroless Ni plating time. Moreover, compared to PF, a new characteristic diffraction peak of 44.5° appears in XRD spectrum of PFN_8_ (Figs. 1b and S10). It can be assigned to the plane (111) of face-centered cubic phase of Ni. Such result confirms the successful deposition of Ni. In addition, the chemical structure of PF and PFN_8_ are characterized by FT-IR (Fig. S2). The characteristic peaks of polyimide can be also observed even after the growth of Fe_3_O_4_ and electroless Ni plating. It confirms that the chemical structure of polyimide is maintained during in-suit growth of Fe_3_O_4_ and electroless Ni plating process. It is noteworthy that a new characteristic peak appears at 557 cm^−1^, which can be attributed to the recognizable Fe–O vibrational band formed between Fe atom and PI-COOH. It provides a strong bonding interaction between Fe_3_O_4_ and PI fibers. As a control, a pure Ni-deposited PI (PN_y_) nonwoven fabric without Fe_3_O_4_ nanoparticles is prepared by oxidative self-polymerization of DA polymerisation, chloroplatinic acid activation, and electroless nickel plating processes. As the electroless Ni plating time is extended to 80 min, uniform Ni element and compact Ni plating can be formed on the surface of PI fibers as shown in Figs. 1h and S8d. As shown in Fig. 1d, the characteristic peak of Ni 2*p* can be observed in XPS spectra of PN_80_. Meanwhile, the high resolution XPS spectra show two characteristic peaks of Ni 2*p* at 855.62 and 873.48 eV, corresponding to Ni 2*p*_3/2_ and Ni 2*p*_1/2_, while the peaks located at 861.06 and 880.18 eV respond to Ni 2*p*_3/2_ satellite and Ni 2*p*_1/2_ satellite, respectively (Fig. S11) [32, 33]. Meanwhile, another binding energy peak appears at 852.11 eV, which is very close to the peak of nickel atom [34]. It indicates that there is metallic nickel attached to PI fibers, which agrees well with the result of XRD in Fig. 1b. In addition, PN_80_ shows excellent thermal stability and highest residual yield of 75.8% due to high content of Ni (Fig. S7). Therefore, it can be concluded that deposited Ni nanoparticles during electroless plating process effectively fill the gaps between Fe_3_O_4_ nanoparticles on PF fiber. As the electroless plating time increased, more Ni nanoparticles can gradually cover the gaps between Fe_3_O_4_ nanoparticles. As a result, a compact and smooth plating with big diameter can be formed for PFN_12_ as shown in Fig. 1g. As an extreme, a compacter and smoother nickel plating can be facilely obtained as displayed in PN_80_ (Figs. 1h and S8d). Subsequently, the tensile strength of PFN_y_ nonwoven fabric is measured and shown in Fig. S12. Notably, compared to PI, the tensile strength of nonwoven fabrics decreases after loading with Fe_3_O_4_ and electroless Ni plating. It can be attributed to the destruction of PI molecular chains by NaOH. Fortunately, the tensile strength of PF, PFN_5_, PFN_8_, PFN_12_, and PN_80_ remains at above 0.7 MPa.

Finally, the deposition of nickel nanoparticles promotes the modulation in permittivity and permeability of PF, thereby regulating its impedance characteristics. Hence, the corresponding Ms of PFN_8_ gradually decreases to 41.68 emu g^−1^ in comparison with that of PF (Fig. 1c), which is still higher than that of PN_80_ (12.67 emu g^−1^). It can be attribute to the fact that the increase content of Ni nanoparticles with weaker magnetic property results in a decrease in Ms of PFN_8_. Meanwhile, the insulation properties of PFN_y_ nonwoven fabric can be simultaneously modulated by controlling the electroless Ni plating time. As a result, the impedance and insulation characteristics of nonwoven fabrics can be readily adjusted and exhibit a tendency to decrease with the prolonging electroless Ni plating time.

### Electromagnetic Parameters and Microwave Absorption of Nonwoven Fabric

**Fig. 2:**
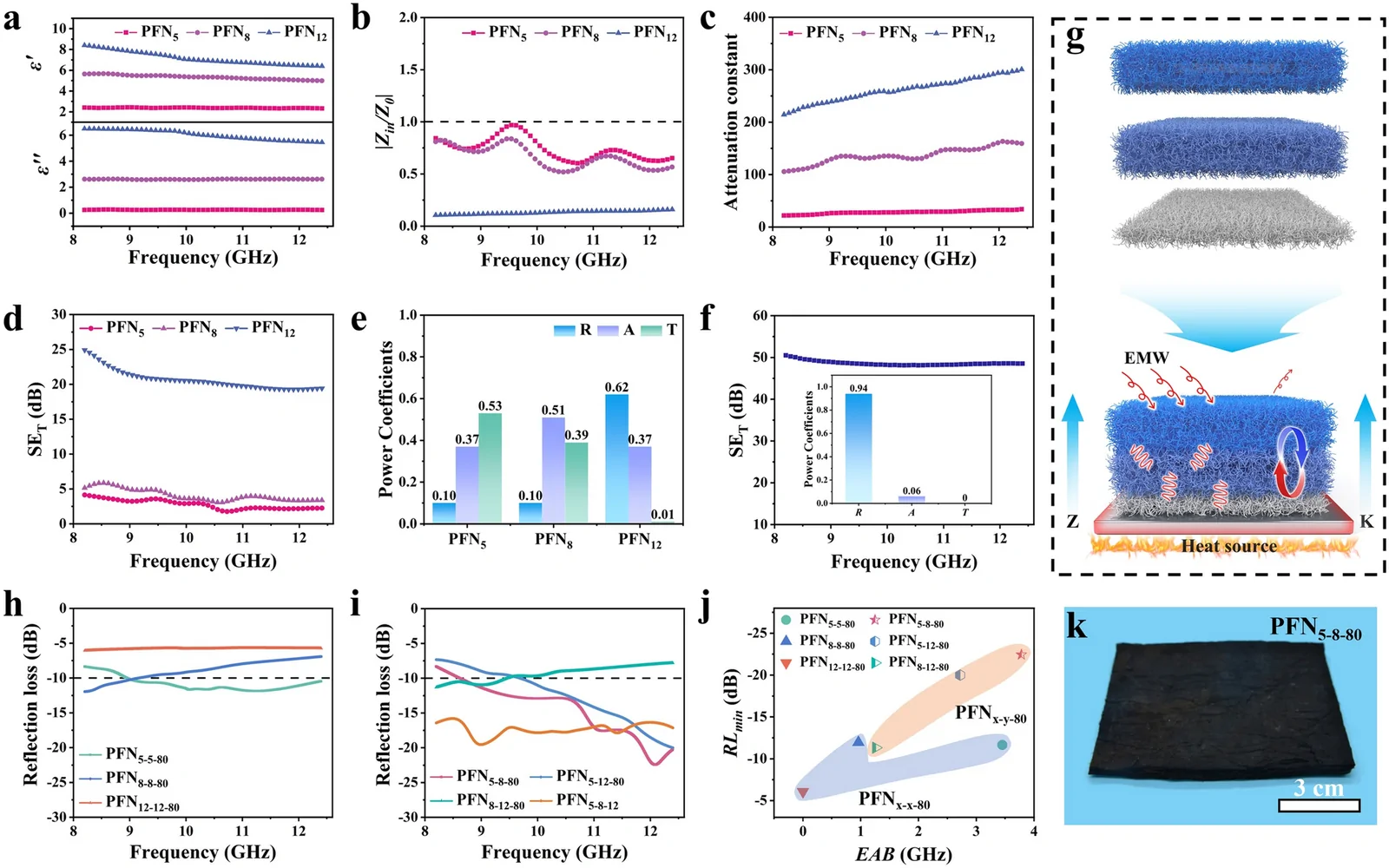
**a** Real permittivity (*ε′*) and imaginary permittivity (*ε′′*), **b** impedance matching (*Z*_*in*_*/Z*_*0*_), **c** Attenuation constant, **d** SE_T_, and **e** Power Coefficients of PFN_5_, PFN_8_, and PFN_12_ at a thickness of 2.5 mm. **f** SE_T_ and Power Coefficients of PN_80_ at a thickness of 0.3 mm. **g** Schematic illustration of the fabrication process of hierarchical polyimide nonwoven fabric with dual-gradient impedance/insulation structure. Reflection loss of **h** PFN_x-x-80_, **i** PFN_x-y-80_ with a thickness of 5.3 mm, and PFN_5-8-12_ with a thickness of 7.5 mm. **j** Comparison in *RL*_*min*_ and *EAB* of PFN_x-x-80_ and PFN_x-y-80_. **k** Optical photograph of PFN_5-8-80_

To explore the MA performance, the complex permittivity (*ε*_*r*_ = *ε′–jε′′*) and permeability (*μ*_*r*_ = *μ′–jμ′′*) of nonwoven fabric are characterized by vector network analyzer (VNA) waveguide method in 8.2–12.4 GHz [35]. The real part of permittivity (*ε′*) and permeability (*μ′*) indicates the storage capacity for electric/magnetic field, whereas the imaginary part (*ε′′* and *μ′′*) represents the loss capacity for electric/magnetic field [36]. As shown in Fig. 2a, the *ε′* of PFN_y_ exhibits a tendency to increase from ~ 2.20 to ~ 5.34, then to ~ 7.16 as electroless Ni plating time extends. Similarly, the corresponding *ε′′* of PFN_y_ displays a tendency to increase from ~ 0.26 to ~ 2.61, and then to ~ 6.03 (Fig. 2a). It can be attributed to the increased content of Ni nanoparticles with high electrical conductivity, thereby endowing it with higher permittivity. It is noting that the *μ′* and *μ′′* display a slight change as the electroless Ni plating time increases (Fig. S13). It may be ascribed to the low content of magnetic nanoparticles and the formation of low-density 3D fluffy network structure in nonwoven fabric. Even so, the controllable evolution of complex permittivity and permeability for PFN_y_ exhibits a promise to become an efficient method for realizing the optimization of impedance matching and attenuation characteristic.

Furthermore, the impedance matching (*|Z*_*in*_*/Z*_*0*_*|*) and attenuation constant (*α*) are the key factor in determining MA performance, which are closely related to complex permittivity and permeability [37]. The corresponding *|Z*_*in*_*/Z*_*0*_*|* of PFN_y_ without metal-plate reflector can be calculated via Eq. S1 at a thickness of 2.5 mm. Moreover, the corresponding *α* of nonwoven fabrics can be simulated by Eq. S2. As display in Fig. 2b, c, PFN_5_ exhibits an excellent impedance matching performance (*|Z*_*in*_*/Z*_*0*_*|*~ 0.74, close to 1), due to the lower content of Ni, allowing abundant EMW penetration into its interior. Unfortunately, PFN_5_ possesses a low *α* ~ 11.83, which is not enough to dissipate those incident EMW, predicting a poor MA performance. As to PFN_8_, the corresponding *|Z*_*in*_*/Z*_*0*_*|* shows a moderate value of ~ 0.66 and higher *α* of 136.47. Its moderate impedance matching and higher attenuation characteristic indicate that PFN_8_ exhibits a promising MA performance. When the electroless plating time increases to 12 min, PFN_12_ demonstrates lowest *|Z*_*in*_*/Z*_*0*_*|* of ~ 0.13 and highest *α* of 261.46. Despite its highest *α*, the poor impedance matching performance leads to a strong reflection of incident EMW on the surface of PFN_12_.

In order to verify the above predictions, EMI shielding effectiveness (EMI SE or SE_T_) and power coefficients of PFN_y_ with the thickness of 2.5 mm are obtained by testing scattering (*S*) parameter and then being calculated by Eqs. S3–S8. As shown in Fig. 2d, e, PFN_5_ exhibits a low SE_T_ of 2.78 dB, low *R* of ~ 0.10, high *A* of ~ 0.37, and higher *T* of ~ 0.53. It indicates that most incident EMW are absorbed and/or transmitted through the composites rather than being reflected. It further confirms that PFN_5_ presents excellent impedance matching performance with exterior air, but the attenuation capacity is weak. Moreover, the corresponding MA performance of PFN_y_ with metal-plate reflector can be calculated by Eqs. S9 and S10 [17, 37]. The effective absorption bandwidth (*EAB*) is considered as the frequency range where *RL* < -10 dB, indicating > 90% of incident EMW is absorbed. As observed in Fig. S14a, d, PFN_5_ exhibits a poor MA with a *RL*_min_ of − 3.83 dB, which is consistent with the above prediction. As the electroless plating time is prolonged to 8 min, the corresponding SE_T_ (4.12 dB) and *A* (0.51) of PFN_8_ display an outstanding increase compared to that of PFN_5_ (Fig. 2d, e). Notably, PFN_8_ displays excellent MA performance (*RL*_min_ = − 64.43 dB, *EAB*_max_ = 4.2 GHz) as shown in Fig. S14b, e, demonstrating its potential as an absorbing layer in impedance gradient structure. When the electroless plating time is further prolonged to 12 min, PFN_12_ exhibits a high SE_T_ of 20.64 dB and *R* of 0.62, decreased *A* of 0.37, and *T* of 0.01. Moreover, PFN_12_ displays a poor MA (*RL*_min_ = − 11.25 dB, *EAB*_max_ = 2.8 GHz) compared to PFN_8_ (Fig. S14c, f). This is mainly attributed to the abominable impedance mismatch caused by excessive Ni nanoparticles, which hinders the absorption of incident EMW. As a control, PN_80_ (0.3 mm) exhibits a similar or even more extreme case. A higher *ε′* (~ 41.39), *ε′′* (~ 11.95) and lower *|Z*_*in*_*/Z*_*0*_*|* (0.03) are shown in Figs. S15 and S16. Hence, a high EMI shielding performance (SE_T_ = 48.63 dB, *R* = 0.94) can be obtained as displayed in Fig. 2f. Therefore, it can be concluded that an excellent impedance matching characteristic is more important than attenuation constant, which can make more incident EMW to be permitted and maximally dissipated in the material.

To optimal MA performance, a hierarchical impedance matching structure can be fabricated by sequentially stacking nonwoven fabrics with different impedance and attenuation characteristic (Fig. 2g). As demonstrated in Fig. S17, a finite element analysis model of PFN_x-y-80_ is simulated by using CST Studio Suite software (PFN_5-8-80_ as an example). In the stimulation, each layer is modeled as a rectangle at the corresponding thickness. The corresponding *RL* of PFN_x-x-80_ are successfully modeled and recorded in Fig. 2h. Among them, PFN_5-5-80_ presents a lowest *RL* of − 11.64 dB and a wide *EAB* of 3.4 GHz (9.0–12.4 GHz), due to the excellent impedance matching with air. Nonetheless, *RL* of composites is decayed as the electroless plating time extends to 8 and 12 min. In detail, PFN_8-8–80_ exhibits higher *RL* and narrow *EAB* (0.96 GHz). As to PFN_12-12-80_, the highest *RL* (~ − 6 dB) arises due to poor impedance matching with air. Such results once again imply that impedance matching is necessary for excellent MA materials. Therefore, PFN_x_ and PFN_y_ combined with PN_80_ are assembled for PFN_x-y-80_. As stimulated in Fig. 2i, j, a remarkable decrease in *RL* appears in PFN_x-y-80_. For PFN_5-8-80_, a conspicuous *RL*_min_ and *EAB* reaches − 22.44 dB and 3.78 GHz, respectively. In this configuration, the sequentially stacking PFN_5_, PFN_8_, and PN_80_ nonwoven fabrics promotes the rational construction of hierarchical gradient structure with more effective impedance matching and attenuation characteristic. Compared to PFN_5-5-80_, the improved electromagnetic dissipation of EMW in PFN_8_ endows PFN_5-8-80_ with an outstanding MA performance. Compared to PFN_12-12-80_ and PFN_8-8-80_, the similar improvement appears in PFN_5-12-80_ (*RL*_min_ = − 20.00 dB, *EAB* = 2.72 GHz) and PFN_8-12-80_ (*RL*_min_ = − 11.32 dB, *EAB* = 1.26 GHz), respectively. It is worthwhile to note that PFN_5-8-80_ possesses the optimal MA performance in comparison with PFN_5-12-80_ and PFN_8-12-80_. It may be ascribed to poor impedance matching between PFN_5_ and PFN_12_ in PFN_5-12-80_ even though PFN_5-12-80_ has a higher attenuation capacity. It leads to a poor MA performance for PFN_5-12-80_. As to PFN_8-12-80_, the existence of PFN_8_ with low impedance characteristic hinders EMW entering the composites, resulting in poor MA performance. Particularly, the MA performance of PFN_5-8-12_ with a thickness of 7.5 mm is simulated and displayed in Fig. 2i. PFN_5-8-12_ exhibits excellent radar stealth performance (*RL*_min_ = − 19.55 dB, *EAB* = 4.2 GHz). However, it is worth noting that PFN_5-8-12_ possesses a greater thickness. Greater thickness restricts its practical application. Hence, PFN_5-8-12_ is not subject to further consideration in system.

As a consequence, it can be concluded that there is a reasonable hierarchical impedance matching structure for PFN_5-8–80_. Hence, for example, a hierarchical impedance structure can be assembled through stacking PFN_5_, PFN_8_, and PN_80_ with gradually decreased impedance characteristic from top to bottom. As shown in Figs. 2k and S18, PFN_5_ (2.5 mm) and PFN_8_ (2.5 mm), and PN_80_ (0.3 mm) are bonded together. The obtained composite exhibits dark front (PFN_5-8-80_) and gray back (PFN_80-8-5_). More importantly, PFN_5-8-80_ possesses an excellent flexibility. As shown in Fig. S19, the large-scale sample (20 × 20 cm^2^) of PFN_5-8-80_ can be rolled into a cylinder. It indicates that the obtained composite can be fitted into complex environments as an excellent flexible material. It can be attributed to the excellent thermal stability of PI (Fig. S7). The PI nonwoven fabric does not undergo obvious thermal decomposition before 400 °C. It implies that the chemical structure of polyimide does not destroy even after treatment at 400 °C. Such excellent thermal and chemical structural stability are important reasons to underpin a fact that PI nonwoven fabric possesses a good flexibility after treatment at 400 °C. In addition, the tensile strength of PFN_5-8-80_ remains at 0.75 MPa even after it is bonded together by spray glue (Fig. S12), which meets the requirements for application in practice. Meanwhile, to investigate the adhesion of Fe_3_O_4_ and Ni to PI fiber, a piece of A4 paper is placed on a thermal stage of 200 °C. PFN_5-8-80_ is pulled from left to right under the pressure of a 100 g weight (Fig. S20). As a result, no obvious exfoliation of Fe_3_O_4_ and/or Ni can be observed on paper. Only the high temperature of 200 °C makes the paper a little yellow. It indirectly suggests that Fe_3_O_4_ and Ni possess strong bonding force with PI fiber at high temperature. Sufficient adhesion between Fe_3_O_4_/Ni and PI fibers is fundamental for their long-term service performance.

### Ultralow-Reflection EMI Shielding Performance of Nonwoven Fabric

**Fig. 3:**
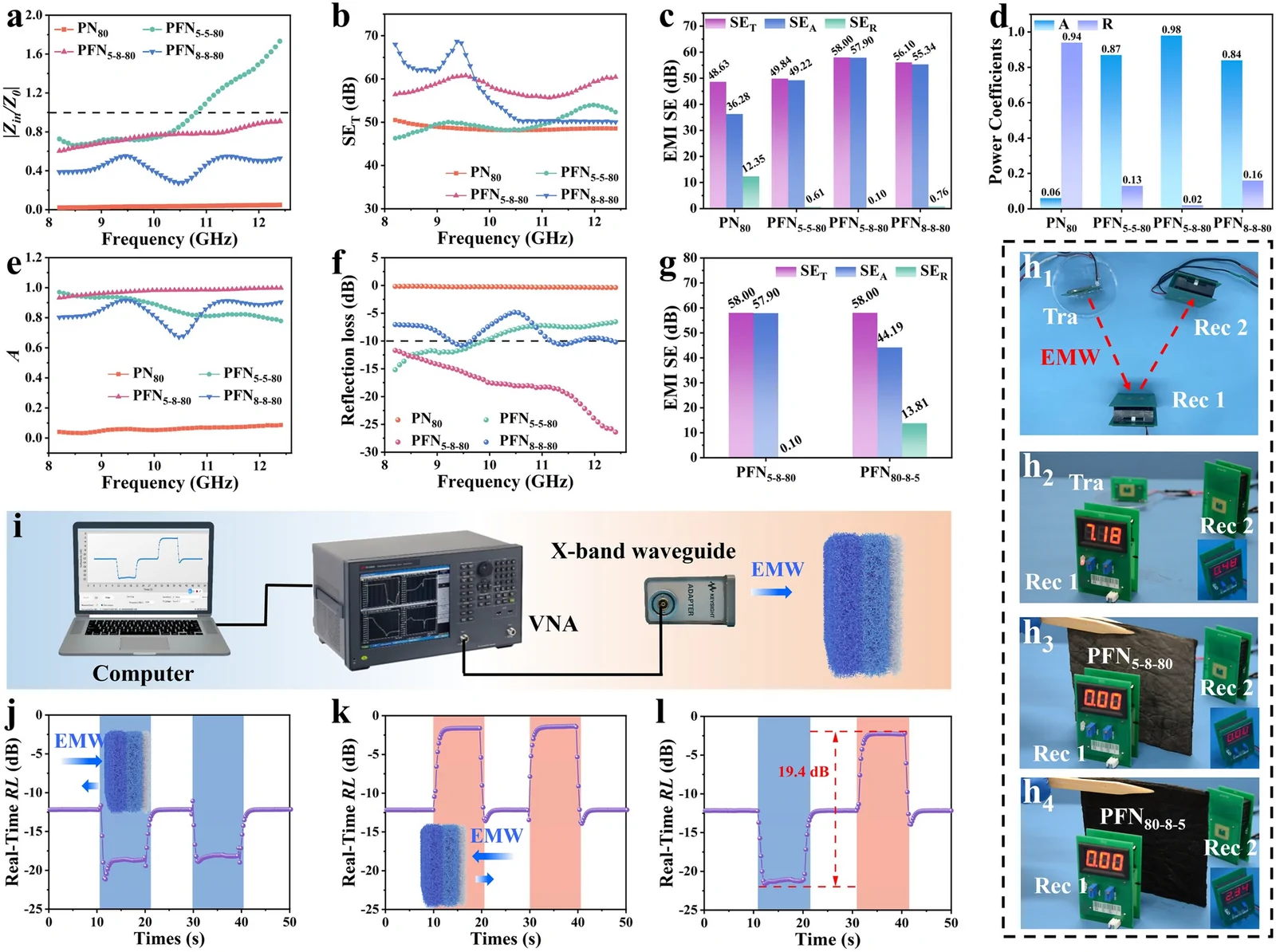
**a** Impedance matching (*|Z*_*in*_*/Z*_*0*_*|*), **b** SE_T_, **c** EMI SE, **d** Power coefficients, **e** *A*, **f** Reflection loss of PN_80_, PFN_5-5-80_, PFN_5-8-80_, and PFN_8-8-80_. **g** EMI SE of PFN_5-8-80_ and PFN_80-8-5_. (**h**_**1**_) Demonstration of EMI shielding system and the changes of EMI shielding system inserted with (**h**_**2**_) nothing, (**h**_**3**_) PFN_5-8-80_, and (**h**_**4**_) PFN_80-8-5_. **i** Schematic diagram of a Real-Time *RL* monitoring system. The changes of Real-Time *RL* monitoring system when the X-band waveguide clamp is faced to **j** PFN_5-8-80_, **k** PFN_80-8-5_, and **l** PFN_5-8-80_ and PFN_80-8-5_, alternately

Meanwhile, the rational construction of hierarchical impedance matching gradient structure endows nonwoven fabric with excellent EMI shielding performance. To clarify the influence of impedance gradient structure on incident EMW, PFN_x-x-80_ and PFN_x-y-80_ are fabricated by a similar approach. And the corresponding impedance matching performance (*|Z*_*in*_*/Z*_*0*_*|*) of PFN_x-x-80_ and PFN_x-y-80_ can be calculated by Eq. S1 [38]. As shown in Fig. 3a, the pure PN_80_ exhibits a lowest *|Z*_*in*_*/Z*_*0*_*|* of ~ 0.03, due to its higher electrical conductivity, indicating impedance mismatch with air that enhances electromagnetic reflection. Therefore, PFN_y_ nonwoven fabrics are adhered with PN_80_ to adjust the impedance matching performance of composite. Compared to PN_80_, |*Z*_*in*_*/Z*_*0*_| of PFN_5-5-80_, PFN_5-8-80_, and PFN_8-8-80_ show a tendency to decrease. Specifically, the curve of |*Z*_*in*_*/Z*_*0*_| for PFN_5-8-80_ exhibits the closest tendency to 1. It indicates that the construction of hierarchical gradient structure in PFN_5-8-80_ endows it with the best impedance matching performance. Subsequently, the final SE_T_, SE_A_, and SE_R_ can be calculated according to Eqs. S3-S8 [39, 40]. As displayed in Fig. 3b, c, the pure PN_80_ possesses a high SE_T_ of 48.63 dB. In addition, PFN_5-8_ exhibits the highest SE_T_ of ~ 7.96 dB (Fig. S21), compared to PFN_5-5_ (~ 2.93 dB) and PFN_8-8_ (~ 6.57 dB). It may be attributed to a fact that the formation of gradient structure endows it with abundant interlayer interfaces between PFN_5_ and PFN_8_, effectively increasing multiple EMW scattering. Therefore, PFN_5-8-80_ presents the highest average SE_T_ of 58 dB, whereas PFN_5-5-80_ and PFN_8-8-80_ exhibit SE_T_ of 49.84 and 56.10 dB, respectively (Fig. 3b, c).

Meanwhile, it is worthy to note that SE_R_ of PFN_5-8-80_ possesses the lowest value of 0.1 dB in comparison with that of PFN_5-5-80_ (0.61 dB) and PFN_8-8-80_ (0.76 dB) (Fig. 3b). In addition, high SE_T_ (> 45 dB) and low SE_R_ (~ 0 dB) are still maintained for PFN_5-8-80_, even over a wider frequency range of 6–40 GHz (Fig. S22). It indicates that fewer EMW reflection arises on the surface of PFN_5-8-80_. To further investigate the differences in EMI shielding mechanism, power coefficients are also calculated and demonstrated in Fig. 3d, e. For PN_80_, it exhibits high *R* (0.94) and low *A* (0.06). It is demonstrated that ~ 94% of incident EMW is reflected and back to the exterior medium, revealing a reflection-dominated EMI shielding mechanism. After being further assembled with PFN, PFN_5-8-80_ exhibits the lowest *R* of ~ 0.02 and highest *A* of ~ 0.98, compared to PFN_5-5-80_ (*R* ~ 0.13,* A* ~ 0.87) and PFN_8-8-80_ (*R* ~ 0.16, *A* ~ 0.84). It suggests that PFN_5-8-80_ possesses an excellent ultralow-reflection EMI shielding performance. For PFN_5-5-80_, PFN_5_ possesses a low electromagnetic parameter (Fig. 2a) and SE_T_ (Fig. 2d), due to the low Ni nanoparticle content. Hence, EMW can easily penetrate PFN_5_, and be sharply reflected on the surface of PN_80_. Importantly, EMW is rarely dissipated in PFN_5_, resulting in a higher *R* of ~ 0.13 and lower *A* of ~ 0.87. As to PFN_8-8-80_, the higher Ni nanoparticle content endows PFN_8_ with higher electromagnetic parameter (Fig. 5a) and SE_T_ (Fig. 2d). In this case, a part of EMW will be reflected and some EMW can enter PFN_8_ to be attenuated. Therefore, a higher *R* of ~ 0.16 and lower *A* ~ 0.84 is obtained. In contrast, for PFN_5-8-80_, excellent impedance matching is formed between PFN_5_ and air, due to the low Ni nanoparticle content of PFN_5_. Therefore, most of EMW can easily enter the interior of PFN_5_. And then EMW can be fully dissipated in PFN_8_. As a result, the lowest *R* of ~ 0.02 and ultrahigh *A* of ~ 0.98 is obtained for PFN_5-8-80_. To further evaluate the dissipation capacity of PFN for EMW, *RL* is calculated by Eq. S11 [41]. As displayed in Fig. 3f, PN_80_ exhibits a high *RL* (~ 0 dB). It implies that almost of incident EMW are reflected on the surface PN_80_. The corresponding average *RL* of PFN_5-5-80_, PFN_5-8-80_, and PFN_8-8-80_ shows a tendency to decrease from ~ − 9.44 to ~ − 17.73 dB, and then increase to ~ − 8.41 dB. More importantly, *RL* of PFN_5-8-80_ is lower than − 10 dB in 8.2–12.4 GHz, which is consistent of the above simulated results of CST (Fig. 2i). It indicates that more than 90% of incident EMW can be absorbed in PFN_5-8-80_. Therefore, PFN_5-8-80_ exhibits a huge potential to be employed as an ultralow-reflection EMI shielding material.

Moreover, PFN_x-y_ can be further assembled with PN_80_ to investigate the effect of different impedance matching gradient structure on electromagnetic performance of PFN_x-y-80_. As shown in Fig. S23, a noticeable difference is demonstrated compared to PFN_5-12-80_, and PFN_8-12-80_. PFN_5-8-80_ exhibits a closer *|Z*_*in*_*/Z*_*0*_*|* to 1. Meanwhile, the SE_T_ of different gradient structure composites demonstrates a minor difference (Fig. S24a). And as shown in Fig. S24b, the corresponding *A* coefficient exhibits a tendency to decrease from 0.98 (PFN_5-8-80_) to 0.87 (PFN_5-12-80_), and then to 0.86 (PFN_8-12-80_). At the same time, similar tendency arises in *RL* (Fig. S24c). PFN_5-8-80_ exhibits the lowest *RL*, which indicates that the least EMW reflection occurs on the surface of PFN_5-8-80_. Overall, optimized hierarchical impedance gradient design enables more EMW enter the materials for dissipation as much as possible, achieving ultralow-reflection EMI shielding. To investigate the effect of impedance gradient structure on EMI shielding performance, EMW incidence direction is controlled. As demonstrated in Figs. 3g and S25, SE_T_ exhibits a similar value of 58 dB, when EMW impinges from PFN_5_ (PFN_5-8-80_) or from PN_80_ (PFN_80-8-5_). Notably, a significant difference appears in SE_R_, SE_A_, *A* and *R*. PFN_5-8-80_ exhibits a high SE_A_ and *A*, while PFN_80-8-5_ displays a low SE_A_ and *A* (Figs. 3g and S26a). Such significant difference can be also observed in *RL* (Fig. S26b,). It once again confirms the effectiveness of impedance matching gradient structure on ultralow-reflection EMI shielding performance.

To visually observe EMI shielding mechanism difference between PFN_5-8-80_ and PFN_80-8-5_, an electromagnetic system (1.0 W, 10.5 GHz) including a transmitter (Tra) and two receivers (Rec 1 and Rec 2) are prepared and displayed in Figs. 3h1 and S27. The numbers of receiver positively correlate with EMW power received from Tra. Upon being emitted by Tra, Rec 1 receives and reflects the incident EMW, which can be detected by Rec 2. As shown in Fig. 3h2, Rec 1 displays a high number of 7.18, when nothing is inserted in the demonstration system. Meanwhile, a smaller number of 0.48 is displayed on Rec 2. Based on the above, PFN_5-8-80_ is inserted between Rec 1 and Rec 2. Importantly, PN_80_ faces to Rec 1, and PFN_5_ faces to Rec 2 (Fig. 3h3 and Movie S1), Rec 1 and Rec 2 rapidly decrease to 0. Contrarily, Rec 1 and Rec 2 exhibits number of 0 and 2.34, respectively, as PFN_80-8-5_ is inserted in the demonstration system (Fig. 3h4 and Movie S2). It indicates that impedance gradient structure formation in PFN_5-8-80_ effectively decrease the reflection and inhibits the transmission of EMW, achieving ultralow-reflection EMI shielding.

In addition, for visual observation of the difference in *RL* for PFN_5-8-80_ and PFN_80-8-5_, a Real-Time reflection loss (*RL*) monitoring system is assembled via computer, VNA, and X-band waveguide clamp (Fig. 3i). A self-programmed software on the computer is employed to control VNA for continuous emission of EMW at 8.2 GHz. As shown in Fig. 3j and Movie S3, a medium *RL* (~ − 12 dB) is displayed in 0–10 s due to the interference of external environment, when no material covers the X-band waveguide clamp. Notably, when PFN_5_ side of PFN_5-8-80_ covers X-band waveguide clamp, the corresponding *RL* sharply decreases to ~ -18 dB in 10–20 s. It shows that PFN_5-8-80_ effectively shields interference of external environment and reduces the reflection of EMW from X-band waveguide clamp. Subsequently, the detected *RL* immediately increased to ~ − 12 dB as PFN_5-8-80_ is removed. And an excellent stability is demonstrated when the PFN_5-8-80_ covers again. The difference in *RL* of 6 dB means that PFN_5-8-80_ can effectively reduce electromagnetic reflection compared to the external environment. As a control, PFN_80-8-5_ repeatedly covers the X-band waveguide clamp. As shown in Fig. 3k and Movie S4, a higher *RL* of ~ − 2 dB can be gained. It implies that PFN_80-8-5_ reflects the incident EMW originating from X-band waveguide clamp. Meanwhile, PFN_5-8-80_ and PFN_80-8-8_ alternately covers the X-band waveguide clamp as shown in Fig. 3l and Movie S5. A lower (~ − 21.5) and higher (~ − 2.1 dB) *RL* can be successively switched. Importantly, the difference in *RL* of 19.4 dB indirectly confirms that PFN_5-8-80_ can achieve a significant reduction in reflection loss. Based on the above, it can be concluded that PFN_5-8-80_ with hierarchical impedance gradient structure possesses a superior low EMW reflection property.

### Radar Stealth Performance of Nonwoven Fabric

**Fig. 4:**
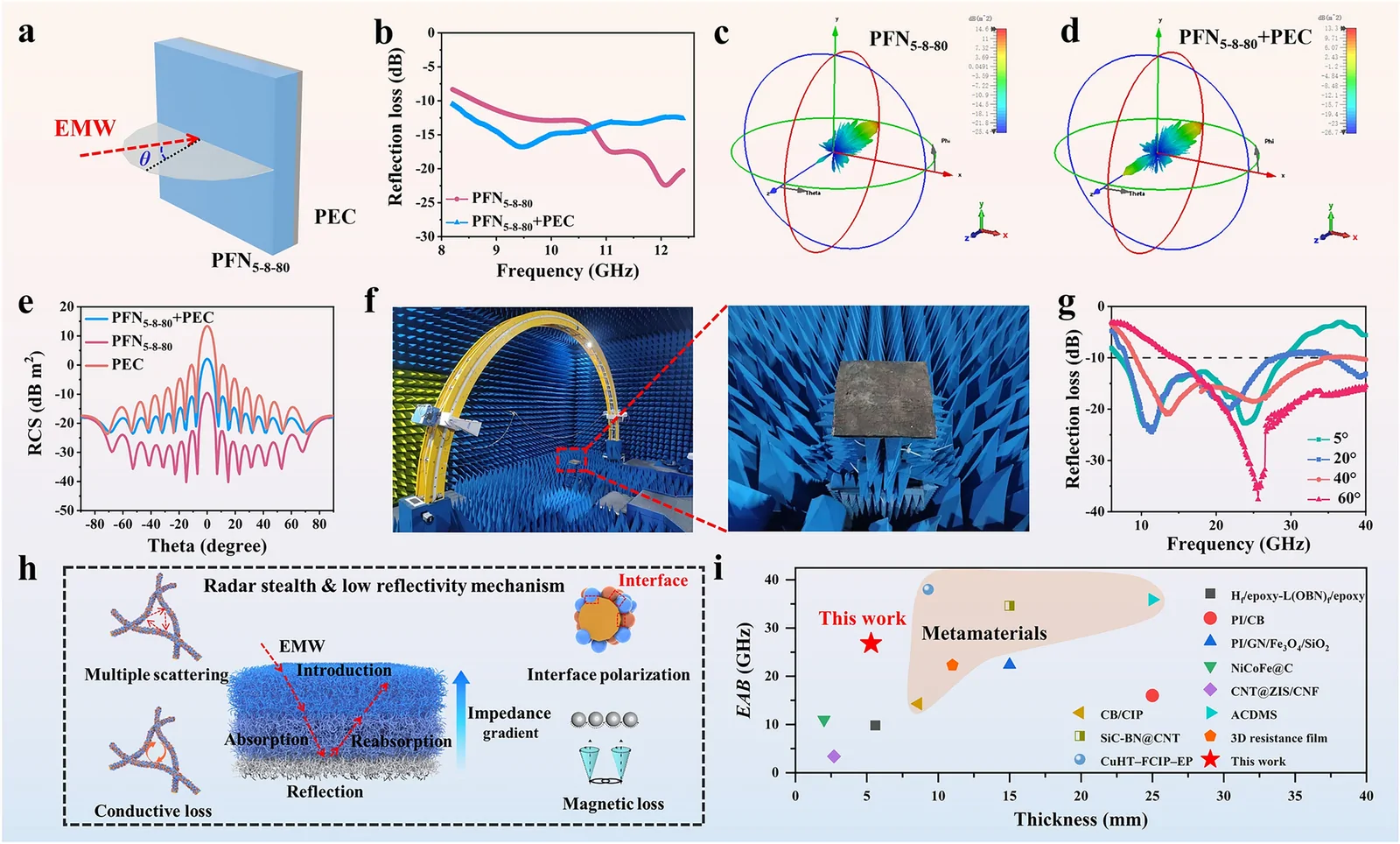
**a** Simulation model of PFN_5-8-80_ + PEC. **b** Reflection loss of PFN_5-8-80_ and PFN_5-8-80_ + PEC simulated by CST. 3D RCS of **c** PFN_5-8-80_, and **d** PFN_5-8-80_ + PEC at 12.4 GHz. **e** 2D RCS of samples at different incidence angles at 12.4 GHz. **f** Photographs of arch method to Reflection loss testing scenes. **g** Reflection loss of PFN_5-8-80_ at different incidence angles of EMW by arch method. **h** Schematic illustration of radar stealth mechanism in nonwoven fabric. **i** Comparing *EAB* and thickness with those reported previously

As we all know, a significant radar exposure usually occurs due to the high electromagnetic reflection property of metal-based military equipment (e.g., warplane and tanks). Therefore, to further investigate the radar stealth performance, PFN_5-8-80_ is placed on a perfect electric conductor (PEC) and modeled for *RL* by CST (Fig. 4a). As shown in Fig. 4b, an excellent *RL* of PFN_5-8-80_ + PEC can be also maintained at − 10.46 ~ − 16.87 dB, compared to that of pure PFN_5-8-80_. Meanwhile, the corresponding *EAB* (4.20 GHz) can covers the entire X-band. To further verify the radar stealth performance, far-field radar cross section (RCS) is simulated and displayed in Figs. 4c, d and S28. In this simulation model, the incidence angle (*θ*) ranges from − 90° to 90° (Fig. 4a). The RCS value of samples is positively correlated with the echo intensity under radar detection [42]. Hence, a low RCS value indicates superior radar stealth performance. As shown in Fig. S28, radar scattered signals at 12.4 GHz exhibit strong reflection due to directly impingement of EMW on PEC surface, inducing a serious radar exposure. As to PFN_5-8-80_, the corresponding radar scattered signals are significantly attenuated (Fig. 4c) due to the excellent microwave performance of PFN_5-8-80_. Similarly, the simulated result of PEC covered by PFN_5-8-80_ (PFN_5-8-80_ + PEC) exhibits dramatic attenuation for radar scattering signals (Fig. 4d). It can be further confirmed by 2D curves of simulated RCS values, as displayed in Fig. 4e. Importantly, the corresponding RCS reduction values of PFN_5-8-80_ + PEC are all greater than 10 dB m^2^ in 8.2–12.4 GHz, compared to PEC (Fig. S29). Based on the above, it indicates that PFN_5-8-80_ possesses strong attenuation characteristic for far-field EMW.

To further validate the radar stealth performance, *RL* at different incidence angles is tested by arch method (Fig. 4f). As shown in Fig. 4g, an excellent radar stealth performance can be achieved at the incidence angles of 5°, 20°, 40°, and 60°. In detail, the *RL*_*min*_ of PFN_5-8-80_ reaches − 23.87 dB at the incidence angle of 5°, while *EAB* can be up to 22.1 GHz (7.1–29.2 GHz), covering C, X, Ku, K, and Ka bands. Importantly, the *RL*_*min*_ of PFN_5-8-80_ increases to a lowest value of − 37.61 dB, and *EAB* is maintained at a high value of 25.6 GHz (14.4–40 GHz) at incidence angle of 60°. Such excellent radar stealth performance can be ascribed to the synergistic effect of impedance gradient structure and multiple attenuation characteristic of PFN_5-8-80_, such as conductive loss of Ni, magnetic loss of Ni and Fe_3_O_4_, interface polarization loss of Ni, Fe_3_O_4_ and PI fiber, multiple scattering between fibers (Fig. 4h). Importantly, to further verify the attenuation mechanism of PFN_5-8-80_, the power loss density (*PLD*) of each layer at 12 GHz is simulated by CST Studio Suite software [43]. As shown in Fig. S30, a dark-blue color displays a lower *PLD* of 5.5 × 10^5^ W m^−3^ for PFN_5_. It indicates that PFN_5_ possesses a poor electromagnetic loss capability. As to PFN_8_, a noticeable green color can be observed, implying a higher *PLD* of 7.2 × 10^6^ W m^−3^. It means that PFN_8_ is adaptive to act as an absorption layer in the gradient structure to dissipate incident EMW. Particularly, a red color and highest *PLD* of 2.2 × 10^7^ W m^−3^ are exhibited in PN_80_. It is mainly attributed to the high electrical conductivity of PN_80_. It causes EMW being strongly attenuated within a thin thickness, resulting in a significant enhancement of *PLD*. Based on its electromagnetic loss capability, the gradient structure is assembled in PFN_5-8-80_, and the corresponding *PLD* is further simulated as shown in Fig. S31. A distinct blue and green areas are presented in PFN_5_ and PFN_8_, respectively. It indicates that incident EMW passes through PFN_5_ with a slight *PLD*, and can be attenuated in PFN_8_ with high *PLD*. Then, the residual EMW further enter and is attenuated at PN_80_ with a highest *PLD*. Finally, PFN_5-8-80_ exhibits an excellent broadband radar stealth performance.

As comparison, recently reported MA materials and their performance are collected in Fig. 4i and Table S1 [44–53]. As summarized, PFN_5-8-80_ presents a significant advantage over the other materials. For instance, Zheng et al. prepare a CNTs@ZIS/CNF aerogel via compositing CNTs@ZIS with CNF, and followed by freeze-drying process [48]. This aerogel exhibits a low density of 0.04 g cm^−3^ and *RL*_*min*_ of − 13 dB at a thickness of 2 mm, due to multiple scattering within the porous structure, and abundant interfacial polarization loss. However, the narrow *EAB* of 3.4 GHz limits its further application in radar stealth. In addition, Ren et al. report a frustum pyramid-shaped SiC-BN@CNT metamaterial by fabricating SiC-BN coated CNTs and subsequently molding through molds [51]. The CST simulation shows that SiC-BN@CNT metamaterial possesses an excellent radar stealth (*RL*_min_ = − 21.07 dB, *EAB* = 34.62 GHz) at big thickness of 15.0 mm. However, the huge thickness significantly reduces the convenience of materials in practice. In this work, PFN_5-8-80_ with a density of 0.15 g cm^−3^ achieves an *EAB* > 22.1 GHz at a small thickness of 5.3 mm. More importantly, the corresponding *RL*_min_ reaches a low value of -23.87 dB. Such excellent comprehensive performance makes it possible for PFN_5-8-80_ as a highly effective radar stealth material with a significant competitive advantage.

### Compatible Radar/Infrared Stealth Performance of Nonwoven Fabric

**Fig. 5:**
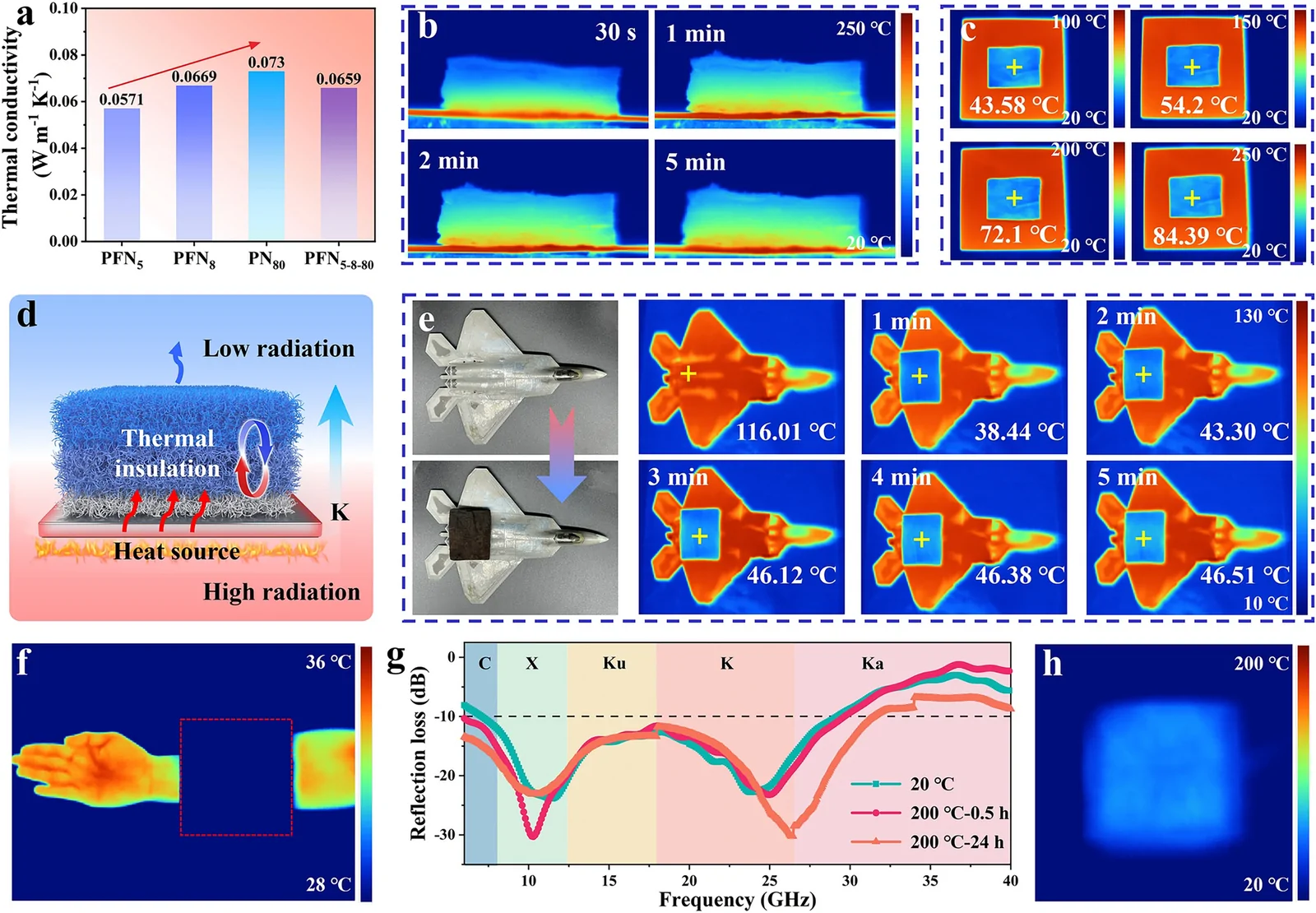
**a** Thermal conductivity of PFN_5_, PFN_8_, PN_80_, and PFN_5-8-80_. **b** Cross-section infrared images of PFN_5-8-80_ on a thermal stage (250 °C) for different times. **c** Infrared images of PFN_5-8-80_ on a thermal stages with different temperature. **d** Infrared stealth mechanism diagram of PFN_5-8-80_. Infrared images of PFN_5-8-80_ on **e** aircraft model for different times and **f** arm. **g** Reflection loss of PFN_5-8-80_ at a thermal stage of 20 °C, 200 °C for 0.5 h, and 200 °C for 24 h. **h** infrared image of PFN_5-8-80_ at a thermal stage of 200 °C for 0.5 h

Low infrared emissity and superior thermal insulation is important for achieving infrared stealth by inhibiting thermal radiation from target [54, 55]. The infrared emissivity of PFN_5_ is measured and displayed in Fig. S32. The PFN_5_ exhibits high infrared emissivity of 87.36%, due to the high emissivity characteristic of Fe_3_O_4_. It indicates that high infrared emissivity is not a significant factor in thermal camouflage performance of PFN_5-8-80_. Noteworthily, the high porosity of PFN_y_ results in the formation of 3D network structure. It effectively restricts the free flow of air within the pores, thereby reducing thermal transfer caused by thermal convection. Meanwhile, the presence of 3D network structure prolongs the thermal conduction path, weakening the thermal conduction of solid phase. Hence, the thermal conductivity (*λ*) is measured to characteristic the thermal insulation capacity (*K*) of samples. As shown in Fig. 5a, the thermal conductivity of sample exhibits a tendency to decrease from 0.073 W m^−1^ K^−1^ (PN_80_) to 0.069 (PFN_8_), and then to 0.0571 (PFN_5_). The obtained thermal conductivity is still located at low value, thereby exhibiting an excellent thermal insulation capacity. Therefore, the thermal insulation gradient can be simultaneously constructed by controlling the thermal conductivity to decrease gradually from top to bottom (Fig. 5d). Benefiting from the construction of thermal insulation gradient structure, a lower average *λ* of 0.0659 W m^−1^ K^−1^ is assigned to PFN_5-8-80_. It plays an important role in inhibiting thermal conduction.

In order to investigate infrared stealth performance, PFN_5-8-80_ is placed on the thermal stage at different temperatures (100–250 °C) and PFN_5_ side faces upward (Fig. S33). Meanwhile, the infrared thermal images of PFN_5-8-80_ are observed and recorded by a thermographer. The cross-section infrared images can be captured to observe the temperature distribution of PFN_5-8-80_ on a thermal stage at 250 °C (Fig. 5b). When PFN_5-8-80_ is placed on the thermal stage, the upper part of sample shows a gradual change from deep blue to light blue, and remained stable after 2 min. Meanwhile, an obvious temperature gradient is clearly observed in cross-section infrared image. It can be ascribed to the successful construction of insulation gradient structure in PFN_5-8-80_. As a result, the upper surface infrared radiation temperature of PFN_5-8-80_ can be stabilized with the ambient environment as shown in Fig. 5c. The infrared radiation temperature of PFN_5-8-80_ is only 84.39 °C, when thermal stage is 250 °C. The infrared imaging color of deep blue on the upper-surface of PFN_5-8-80_ is close to that of background. The area covered by PFN_5-8-80_ can be well hidden in infrared thermal imaging. It suggests that PFN_5-8-80_ with excellent insulation gradient structure effectively resists thermal radiation derived from high-temperature source, thereby achieving an outstanding infrared stealth performance. More importantly, the excellent long-term stability in infrared stealth performance successfully confirms the formation of strong interfacial interaction between Fe_3_O_4_, Ni and PI fiber. It accelerates PFN_5-8-80_ to resist stress originated from high-temperature source of 250 °C. To further verify the environment adaptability, the infrared stealth performance of PFN_5-8-80_ in different environments is conducted. As displayed in Fig. 5e, PFN_5-8-80_ is placed on aircraft models to simulate infrared stealth performance for military equipment. After 1 min, the infrared radiation temperature of PFN_5-8-80_ decreases from 116.01 to 38.44 °C. A low infrared radiation temperature (~ 46.51 °C) is maintained even after 5 min. In addition, PFN_5-8-80_ exhibits an excellent thermal camouflage performance for the arm, presenting a consistent deep-blue color compared to its surrounding (Fig. 5f).

**Fig. 6:**
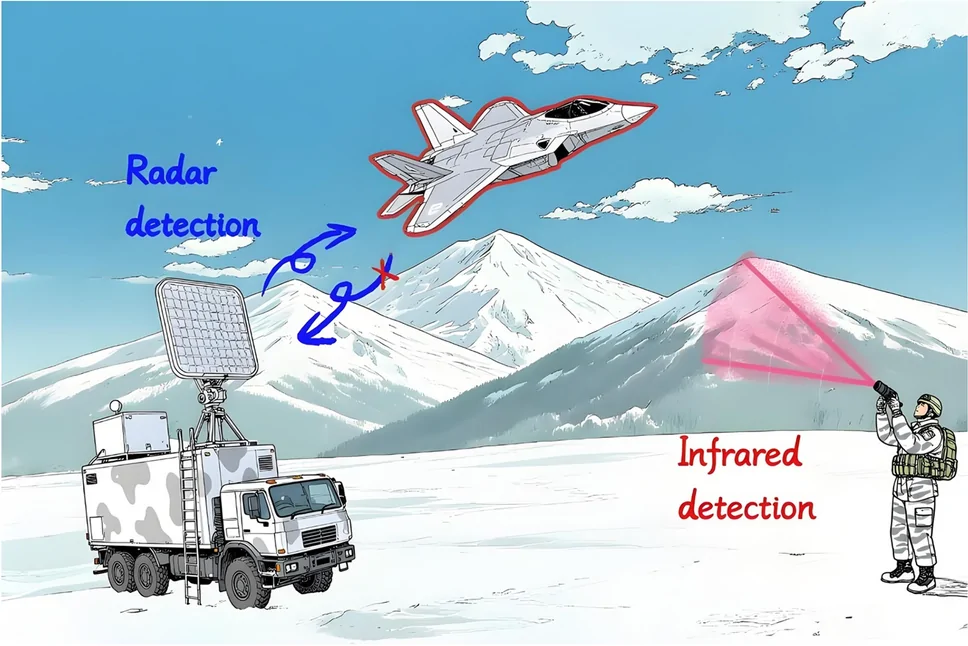
The possible application scenario of PFN_5-8-80_ with compatible high-temperature resistant radar/infrared stealth performance

To demonstrate the compatible high-temperature resistant radar/infrared stealth performance, PFN_5-8-80_ is placed on a thermal stage of 200 °C. The radar and infrared stealth performance are measured by arch method and infrared thermographer, respectively. As shown in Fig. 5g, an excellent radar stealth performance can be maintained at the thermal stage of 200 °C for 0.5 h. Importantly, its *EAB* shows excellent enhancement from 22.1 to 23.6 GHz, as the temperature increases from 20 to 200 °C. The enhanced *EAB* covers C, X, Ku, K, and Ka bands. Additionally, such excellent high-temperature resistant radar stealth performance once again confirms the formation of strong interfacial bonding between Fe_3_O_4_ and Ni nanoparticle and PI fiber. At the same time, an outstanding in situ infrared stealth performance for 200 °C can be achieved as shown in Fig. 5h. To further verify the thermal stability of PFN_5-8-80_, it is placed on a thermal stage at 200 °C. And the thermal treatment time is accumulated for 24 h. Subsequently, the radar and infrared stealth performance are measured by arch methond and infrared thermorgrapher, respectively. As shown in Fig. 5g, an excellent radar stealth performance is maintained, even after PFN_5-8-80_ is treated at 200 °C for 24 h. Meanwhile, it still displays a superior infrared stealth performance in Fig. S34. Based on the above, it can be concluded that PFN_5-8-80_ possesses an excellent stability in radar/infrared stealth performance, due to the oxidation resistance of Fe_3_O_4_ and Ni. It verifies that compatible high-temperature resistant radar/infrared stealth performance can be successfully realized in this system. As a consequence, such excellent compatible radar/infrared stealth performance makes it promising for PFN_5-8-80_ to be applied in the fields of military camouflage under high-temperature scenario (as warplane, Fig. 6).

## Conclusions

In summary, a novel conductive/magnetic PI nonwoven fabric is prepared by alkali treatment, Fe^3+^ ion exchange, thermal reduction, and electroless Ni plating process. The carboxyl group originated from hydrolysis of imide rings in PI molecular served as strong bonding anchors for Fe_3_O_4_ nanoparticles generated by Fe^3+^ ion exchange and thermal reduction. Meanwhile, PDA roughens fiber can promote the in situ immobilization/reduction of Pt^4+^ to Pt nanoparticle, thus facilitating Ni deposition during electroless Ni plating. As a consequence, the impedance/insulation characteristics of PFN_y_ can be easily adjusted by controlling the in situ growth of Fe_3_O_4_ and electroless nickel plating process. A new strategy of constructing hierarchical dual-gradient impedance/insulation structure is implemented to achieve EMI shielding, radar and infrared stealth via stacking PFN_y_ with gradually decreased impedance/insulation characteristic from top to bottom. The formation of impedance gradient structure induces more EMW to enter the composite and be dissipated as much as possible, endowing the composite with an outstanding EMI shielding (SE_T_ > 45 dB in 6–40 GHz) and radar stealth (*RL*_min_ = − 23.87 dB, *EAB* > 22.1 GHz) performance. Meanwhile, the construction of thermal insulation gradient structure can endow it with a low thermal conductivity (0.0659 W m^−1^ K^−1^), bringing an excellent infrared stealth performance. Importantly, the strong interfacial interactions between Fe_3_O_4_, Ni and PI fiber enable PFN_y_ to resist stress from high-temperature heat source (200 °C), achieving a compatible high-temperature resistant radar/infrared stealth performance. Such excellent radar/infrared stealth performance of nonwoven fabrics endows it with a broad application prospect in the fields of military camouflage under high-temperature conditions.

## Supplementary Information

Below is the link to the electronic supplementary material.

Supplementary file 1 (MP4 229 kb)

Supplementary file 2 (MP4 201 kb)

Supplementary file 3 (MP4 899 kb)

Supplementary file 4 (MP4 438 kb)

Supplementary file 5 (MP4 1188 kb)

Supplementary file 6 (DOCX 22927 kb)
