# Supplementary material for: Dual-Gradient Impedance/Insulation Structured Polyimide Nonwoven Fabric for Multi-Band Compatible Stealth
Source: Nanomicro Lett. 2026 Jan 4;18:130. doi: 10.1007/s40820-025-01966-z (PMC12764743; doi:10.1007/s40820-025-01966-z)
Supplement: Supplementary file 6 — (DOCX 22927 kb) [file 40820_2025_1966_MOESM6_ESM.docx]

Supporting Information for

**Dual-Gradient Impedance/Insulation Structured Polyimide Nonwoven Fabric for Multi-Band Compatible Stealth**

Xinwei Tang^1^, Wei Hong^1^, Hongmiao Gao^1^, Shuangshuang Li^1^, Wei Li^1^, Kaixin Lai^1^, Mingzhen Xu^2^, Zaiyin Hu^3^, Yan Li^4^, Zicheng Wang^1^* and Tianxi Liu^1^*

^1^ The Key Laboratory of Synthetic and Biological Colloids, Ministry of Education, School of Chemical and Material Engineering, International Joint Research Laboratory for Nano Energy Composites, Jiangnan University, Wuxi, Jiangsu 214122, P. R. China

^2^ Yangtze Delta Region Institute (Huzhou), University of Electronic Science and Technology of China, Huzhou, Zhejiang 313001, P. R. China

^3^ Guizhou Aerospace Wujiang Electro-Mechanical Equipment Co., Ltd., No. 20-5, Dalian Road Aerospace Industrial Park, Huichuan District, Guizhou, Zunyi 563100, P. R. China

^4^ Jiangsu Ferrotec Semiconductor Technology Co., Ltd. Yancheng, Jiangsu 214000, P. R. China

*Corresponding author. E-mail: [wangzc@jiangnan.edu.cn](mailto:wangzc@jiangnan.edu.cn) (Zicheng Wang); [txliu@jiangnan.edu.cn](mailto:txliu@jiangnan.edu.cn) (Tianxi Liu)

**S1 Experimental Section**

**S1.1 Materials**

Tris (hydroxymethyl) aminomethane (Tris, ≥99%), 3-Hydroxytyramine hydrochloride (DA, ≥99%), Dimethylaminoborane (DMAB, ≥97%), Chloroplatinic acid hexahydrate (H_2_PtCl_6_·6H_2_O, ≥99.9%), Lactic acid (≥90%) and Iron(III) Chloride Hexahydrate (FeCl_3_·6H_2_O, ≥99%) were purchased from Adamas Reagent Co., Ltd. Nickel (Ⅱ) sulfate hexahydrate (NiSO_4_·6H_2_O, ≥98.5%); Trisodium citrate dehydrate (≥99.5%), Hydrochloric acid (HCl, 36.0～38.0%) and Sodium hydroxide (NaOH, ≥96%) were purchased from Sinopharm Chemical Reagent Co., Ltd. 75 spray-glue was purchased from Minnesota Mining and Machinery Company. All other chemicals were used as received without further purification.

**S1.2 Preparation of Fe_3_O_4_/Ni-loaded polyimide nonwoven fabrics**

Firstly, a PI nonwoven fabric was prepared according to our previously reported method [26]. Subsequently, the nonwoven fabric was immersed in a 10 wt% NaOH solution for 3 min. Afterwards, it was sufficiently washed by Deionized (DI) water to remove residual NaOH. Then, the sample was placed in a 37 wt% solution of FeCl_3_·6H_2_O at 80 °C for 5 h. The treated nonwoven fabric was further washed by anhydrous ethanol, and then placed in an oven at 80 °C for 2h. Finally, the dried PI nonwoven fabric was thermal treated at 400 °C for 4 h under 12 vol% hydrogen-argon atmosphere. As a result, magnetic nanoparticle-loaded PI nonwoven fabric (PI@Fe_3_O_4_) was obtained, which can be named as PF.

Secondly, PF nonwoven fabric was placed in 10 mM Tris and 2 g L^-1^ DA solution, and reacted under magnetic stirring for 24 h. After the reaction, the nonwoven fabric was sufficiently washed by DI water. And then, it was further immersed in 0.5 g L^-1^ H_2_PtCl_6_ solution for 0.5 h. Afterwards, the nonwoven fabric was fully washed with DI water, and placed in a electroless nickel (Ni) plating solution for y min, which can be marked as PFN_y_.

As a control, a pure Ni-deposited PI (PN_y_) nonwoven fabric without Fe_3_O_4_ nanoparticles was similarly prepared by oxidative self-polymerization of DA, chloroplatinic acid activation, and electroless nickel plating processes. In detail, PI nonwoven fabric was placed in 10 mM Tris and 2 g L^-1^ DA solution, and reacted under magnetic stirring for 24 h. After the reaction, the nonwoven fabric was sufficiently washed by DI water. And then, it was further immersed in 0.5 g L^-1^ H_2_PtCl_6_ solution for 0.5 h. Afterwards, the nonwoven fabric was fully washed with DI water, and placed in a electroless Ni plating solution for y min. The obtained nonwoven fabric was labeled as PN_y_.

Electroless nickel plating process was carried out in a plating solution containing a 4:1 vol ratio of solution A and solution B. Solution A can be obtained by quantitatively preparing a solution mixture of NiSO_4_·6H_2_O (80 g L^-1^) and Trisodium citrate dehydrate (40 g L^-1^). Solution B was freshly prepared by dissolving 3 g L^-1^ DMAB and adjusting pH = 7 with ammonia.

**S1.3 Fabrication of dual-gradient polyimide composite nonwoven fabrics**

The dual-gradient polyimide nonwoven fabric was fabricated by combining with different PFN_y_ and PN_y_ nonwoven fabrics. Finally, the nonwoven fabrics were pasted by 75 spray-glue, which was denoted as PFN_x-y-z_.

**S1.4 Characterization**

The morphology of specimens was observed by a field emission scanning electron microscope (FE-SEM, Hitachi, S4800, Japan) at an accelerating voltage of 3 kV. Energy dispersive X-ray spectroscopy (EDS) analysis was also performed to analyze the surface composition of specimens. X-ray diffraction (XRD) patterns were characterized on a Bruker D2 X-ray diffractometer with a Cu K*α* X-ray source (*λ* = 1.5418 Å). X-ray photoelectron spectroscopic (XPS) was conducted on an ESCA 2000 (VG Microtech, UK) by using a monochromic Al *Kα* X-ray source. Fourier transform infrared spectroscopy (FTIR) was performed using a Nicolet 6700 USA spectrophotometer. Thermal gravimetric analysis (TGA) was carried out using a TGA/DSC1/1100SF system under a N_2_ atmosphere at a heating rate of 10 °C/min. The stress-strain curves of samples were tested with an electronic universal testing machine (UTM2203, Shenzhen Suns Technology Stock Co. Ltd., China). Polyimide nonwoven fabrics were prepared by electrostatic spinning machine (ET-2535H, Beijing Ucalery Co.,Ltd., China). The thermal conductivity of nonwoven fabric was measured by using the transient plane source method on a hot disk instrument (TPS2500S, Hot Disk Inc., Sweden). Using Agilent E5234B vector network analyzer and waveguide method, the complex permittivity (*ε_r_=ε′-jε′′*), permeability (*μ_r_=μ′-jμ′′*) and scattering parameters (S_11_ and S_21_) of nonwoven fabrics were measured in the frequency of 8.2-12.4 GHz. Subsequently, the *|Z_in_/Z_0_|* of PFN_y_ without metal-plate reflector and attenuation constant (*α*) was calculated by Eqs. S1, S2

$\left| \text{z}_{\text{in}}\text{/}\text{z}_{\text{0}} \right|\text{=}\left| \sqrt{\frac{\left( \text{1+}\text{S}_{\text{11}} \right)^{\text{2}}\text{-}{\text{S}_{\text{21}}}^{\text{2}}}{\left( \text{1-}\text{S}_{\text{11}} \right)^{\text{2}}\text{-}{\text{S}_{\text{21}}}^{\text{2}}}} \right|$ (S1)

$\text{α}=\frac{\sqrt{\text{2}}\text{π}\text{f}}{\text{c}}\sqrt{\left( \text{μ}^{\text{''}}\text{ε}^{\text{''}}-\text{μ}^{\text{'}}\text{ε}^{\text{'}} \right)\text{+}\sqrt{\left( \text{μ}^{\text{''}}\text{ε}^{\text{''}}\text{-}\text{μ}^{\text{'}}\text{ε}^{\text{'}} \right)^{\text{2}}\text{+}\left( \text{μ}^{\text{''}}\text{ε}^{\text{'}}\text{+}\text{μ}^{\text{'}}\text{ε}^{\text{''}} \right)^{\text{2}}}}$ (S2)

, where *f* is the frequency, and *c* is the speed of light under vacuum conditions.

Meanwhile, EMI SE was measured on a vector network analyzer (Agilent E5234B) by using waveguide method in frequency range of 8.2-12.4 GHz. The corresponding EMI SE (SE or SE_T_) and impedance matching performance (|*Z_in_/Z_0_*|) can be obtained by calculating the scattering parameters (S_11_ and S_21_) as follow Eqs. S3-S8.

$\text{ }\text{R=}{\text{|}\text{S}_{\text{11}}\text{|}}^{\text{2}}\text{=}{\text{|}\text{S}_{\text{22}}\text{|}}^{\text{2}}$ (S3)

$\text{ }\text{T=}{\text{|}\text{S}_{\text{21}}\text{|}}^{\text{2}}\text{=}{\text{|}\text{S}_{\text{12}}\text{|}}^{\text{2}}$ (S4)

$\text{ }\text{A=1-R-T}$ (S5)

$\text{SE}_{\text{R}}\text{=-10}\log\left( \text{1-R} \right)\text{=-10}\log\left( \text{1-}\left| \text{S}_{\text{11}} \right|^{\text{2}} \right)$ (S6)

$\text{SE}_{\text{A}}\text{=-10}\log\left( \frac{\text{T}}{\text{1-R}} \right)\text{=-10log(}\frac{\left| \text{S}_{\text{21}} \right|^{\text{2}}}{\text{1-}\left| \text{S}_{\text{11}} \right|^{\text{2}}}\text{)}$ (S7)

${\text{ }\text{SE}}_{\text{T}}\text{=}\text{SE}_{\text{R}}\text{+}\text{SE}_{\text{A}}\text{+}\text{SE}_{\text{M}}$ (S8)

, when the SE_T_ exceeded 10 dB, SE_M_ can be generally negligible.

The microwave absorption performance of samples can be described by the reflection loss (*RL*) values, and be calculated by Eqs. S9, 10:

$\text{Z}_{\text{in}}\text{=}\text{Z}_{\text{0}}\sqrt{\frac{\text{μ}_{\text{r}}}{\text{ε}_{\text{r}}}\text{tanh}\left[ \text{j}\left( \frac{\text{2πfd}}{\text{c}} \right)\sqrt{\text{ε}_{\text{r}}\text{μ}_{\text{r}}} \right]}$ (S9)

$\text{RL (dB)}\text{=}\text{20lg}\left| \frac{\text{Z}_{\text{in}}\text{-}\text{Z}_{\text{0}}}{\text{Z}_{\text{in}}\text{+}\text{Z}_{\text{0}}} \right|$ (S10)

, where *Z_in_* and *Z_0_* represented the input impedance of sample and the impedance under vacuum conditions, respectively, and *d* is the thickness of sample.

Moreover, *RL* (dB) was also calculated by Eq. S11 to evaluate the ability of electromagnetic waves reflected from the surface of nonwoven fabrics.

$\text{ RL}\text{=10logR=20}\log\text{|}\text{S}_{\text{11}}\text{|}$ (S11)

Furthermore, a self-made electromagnetic wave transmitter-receiver (10.5 GHz, 1W) was carried out to demonstrate EMI shielding and absorption performance of samples. Reflectivity of samples (200 mm*200 mm*5.3 mm) are also measured on vector network analyzers (Rohde & Schwarz, ZNA43. Ceyear, 3671G) by using arch method in 6-40 GHz. The nonwoven fabrics were further placed on a thermal stage of 200 ^o^C (Shenzhen Jinglianghe Technology Co., Ltd.) to evaluate their high-temperature radar stealth performance. Meanwhile, the infrared thermal imager (FLUKE Ti400+, Fluke Corporation, USA) was applied to observed and recorded the changed in infrared radiation of samples.

**S2 Supplementary Figures and Tables**

**
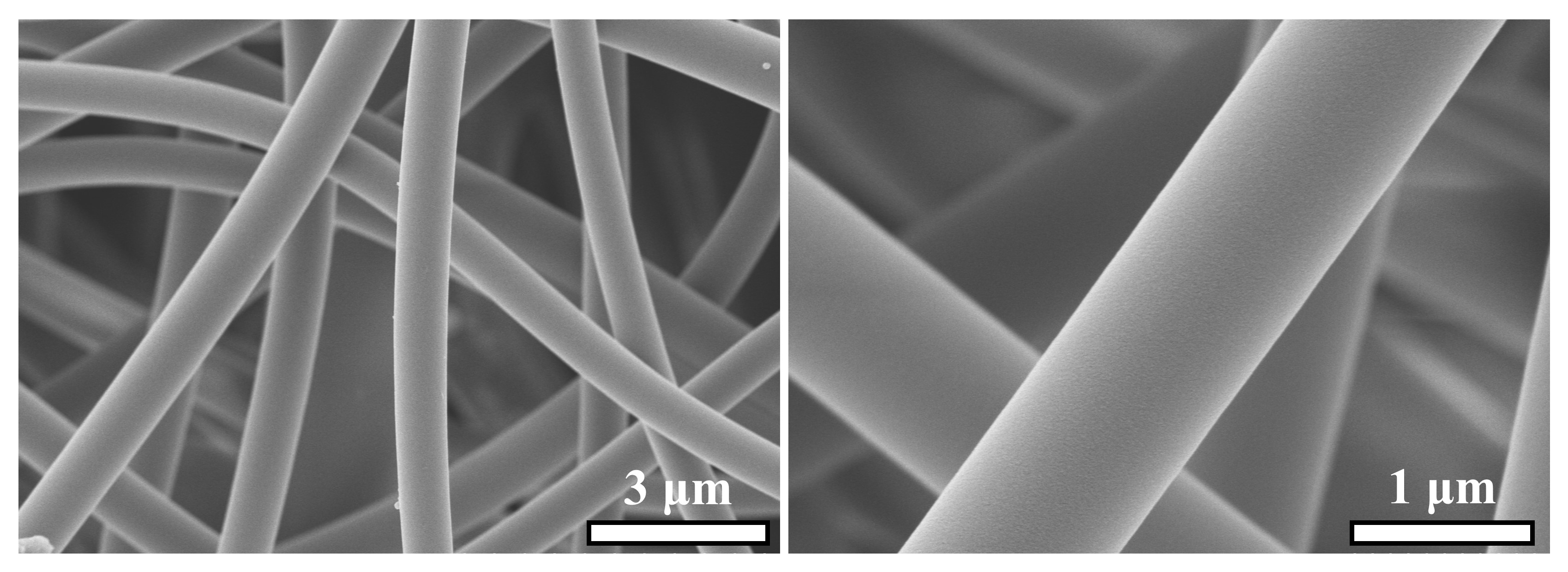
**

**Fig. S1** SEM images of PI nonwoven fabric


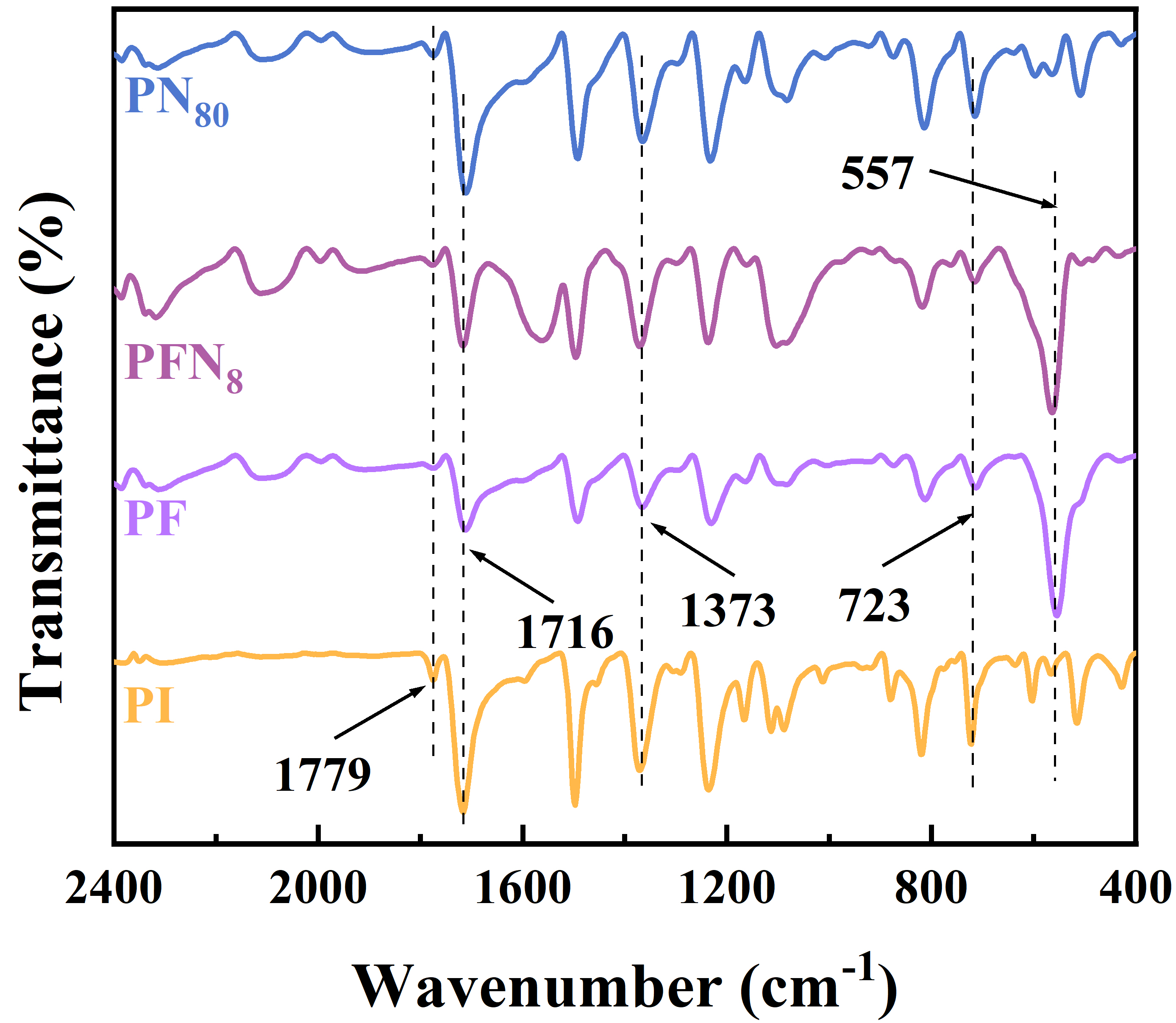


**Fig. S2** FT-IR curves of PI, PF, PFN_8_, and PN_80_ nonwoven fabrics


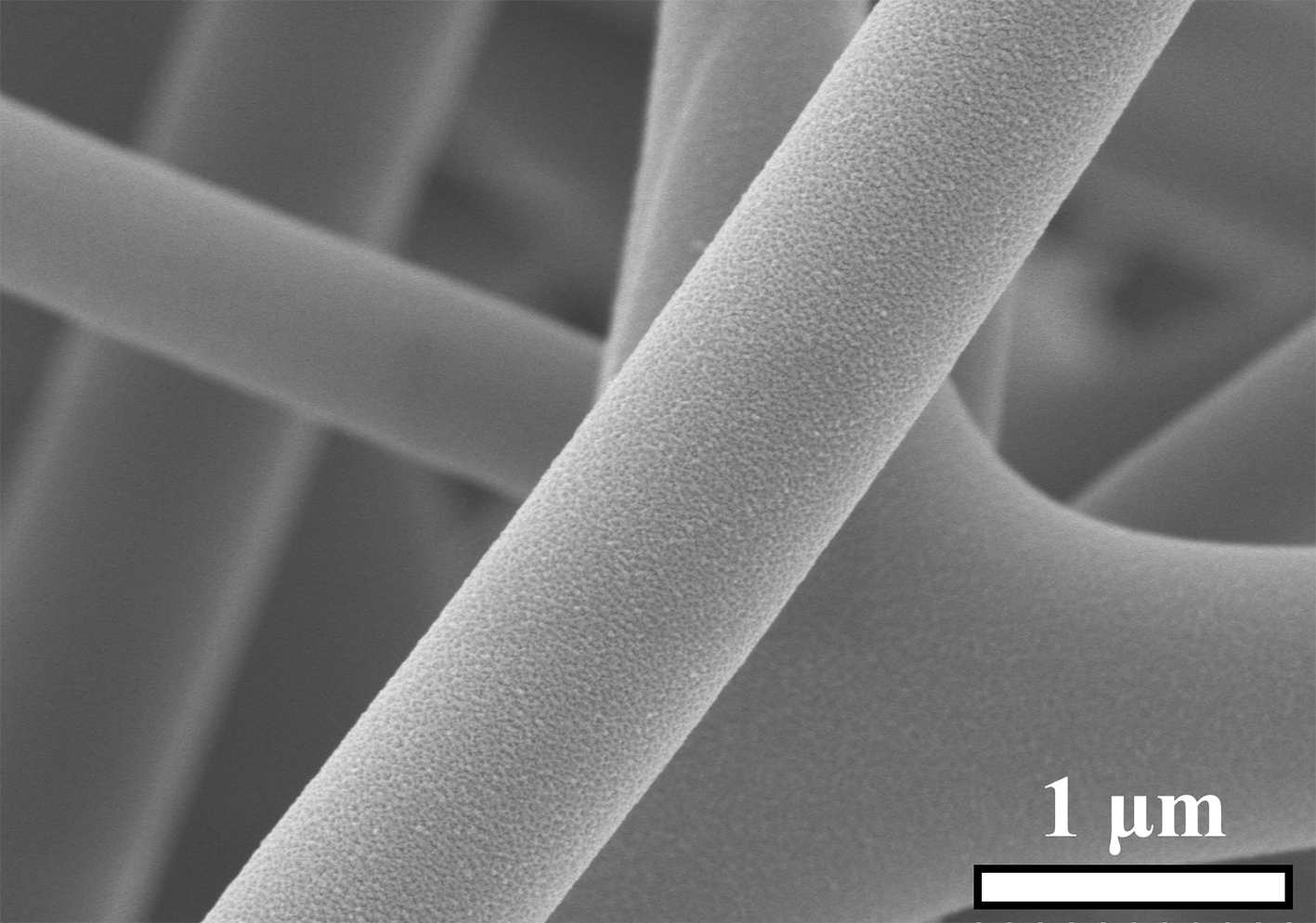


**Fig. S3** SEM image of NaOH-treated PI nonwoven fabric

**
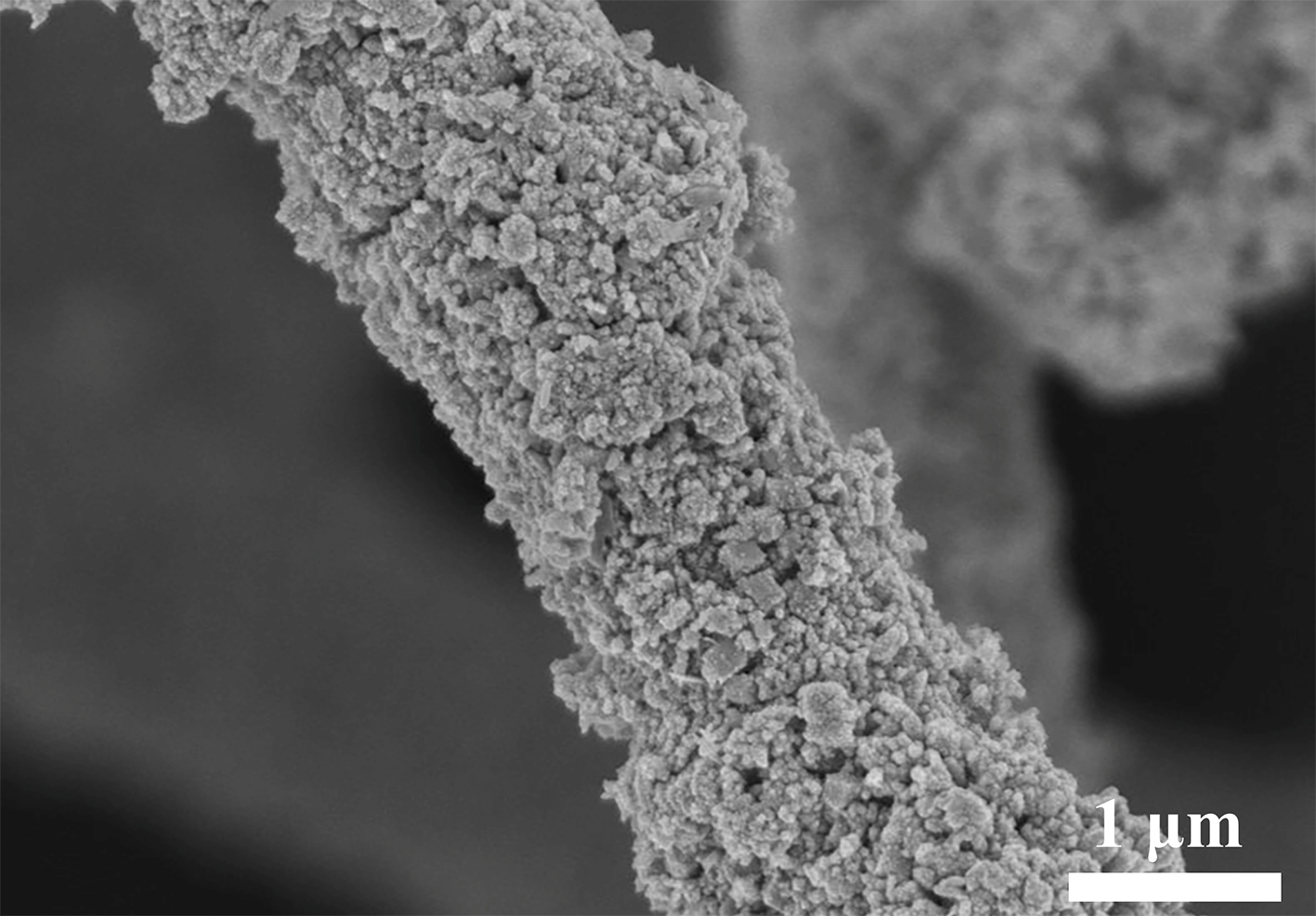
**

**Fig. S4** SEM image of PF nonwoven fabric

**
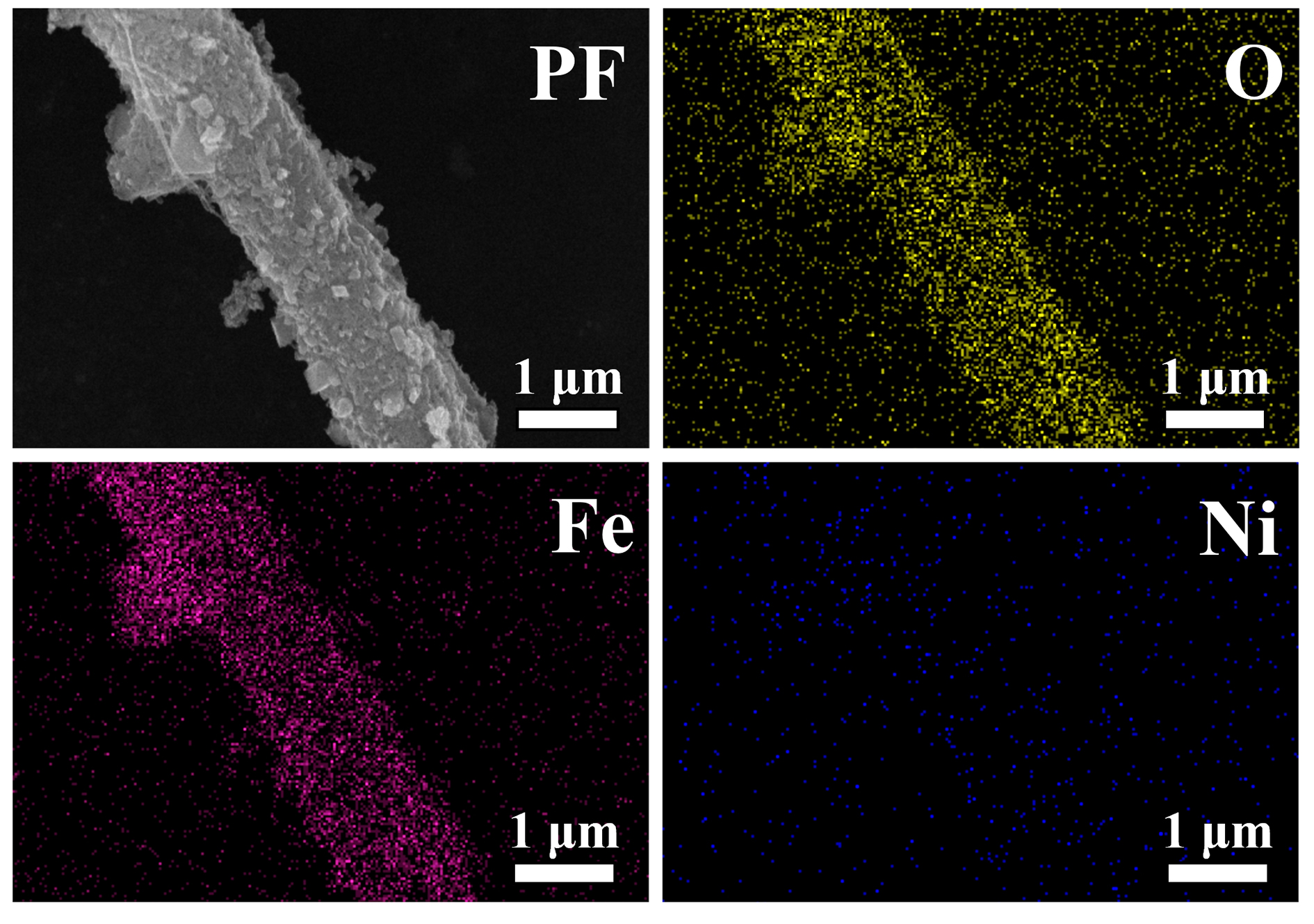
**

**Fig. S5** EDS mapping images of PF nonwoven fabric


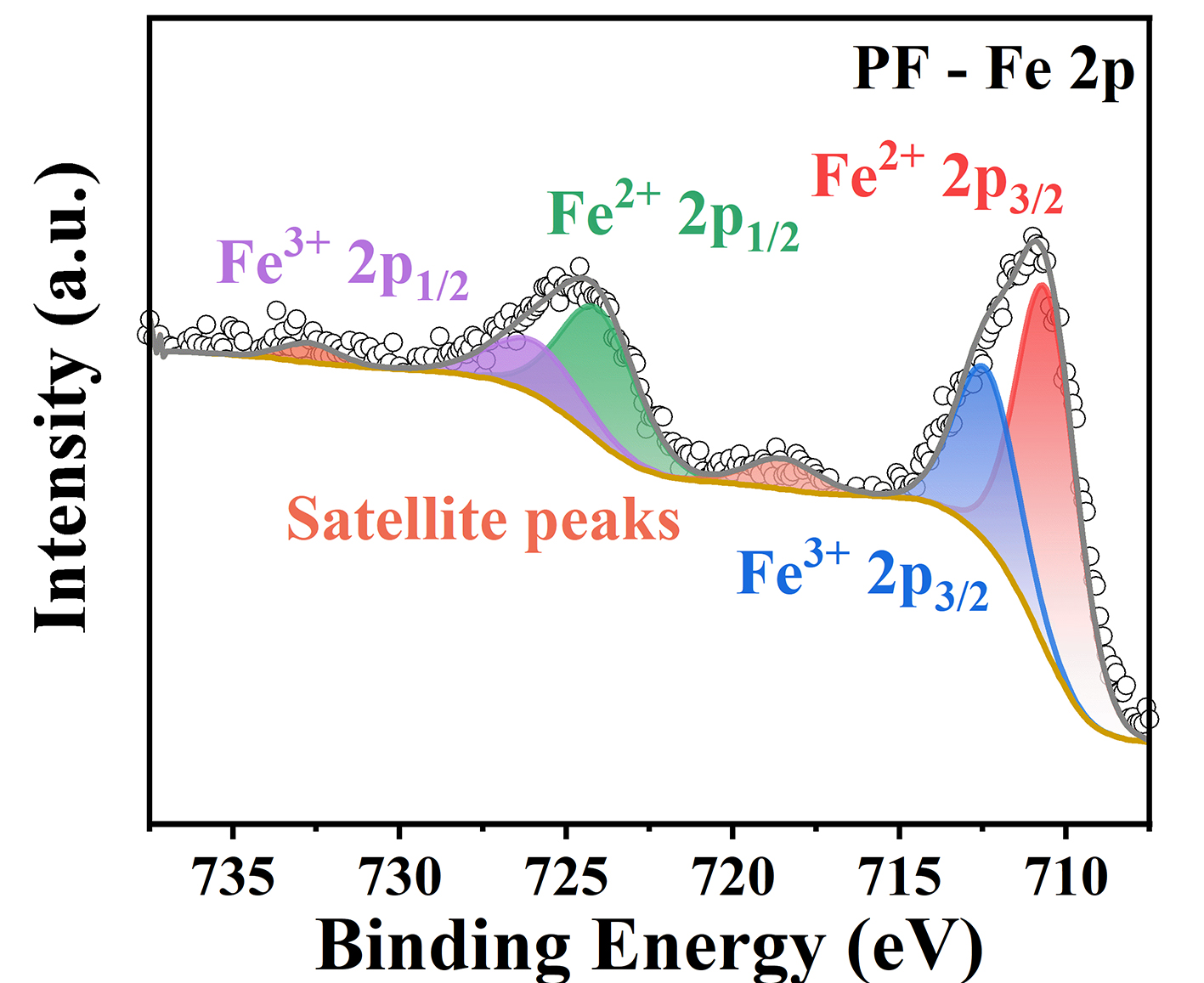


**Fig. S6** High resolution XPS spectrum of Fe 2p for PF


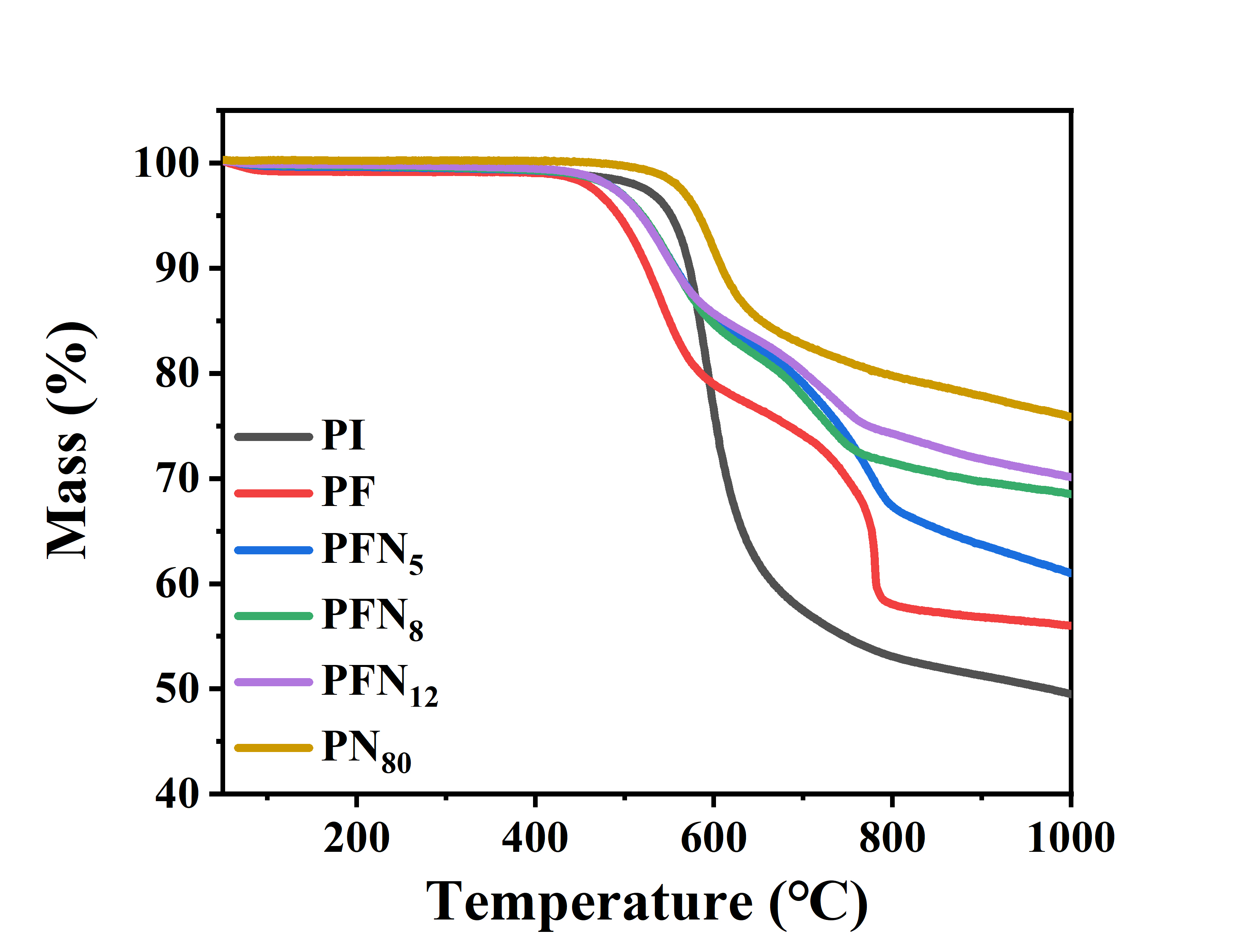


**Fig. S7** TGA curves of PI, PF, PFN_5_, PFN_8_, PFN_12_, and PN_80_ nonwoven fabrics


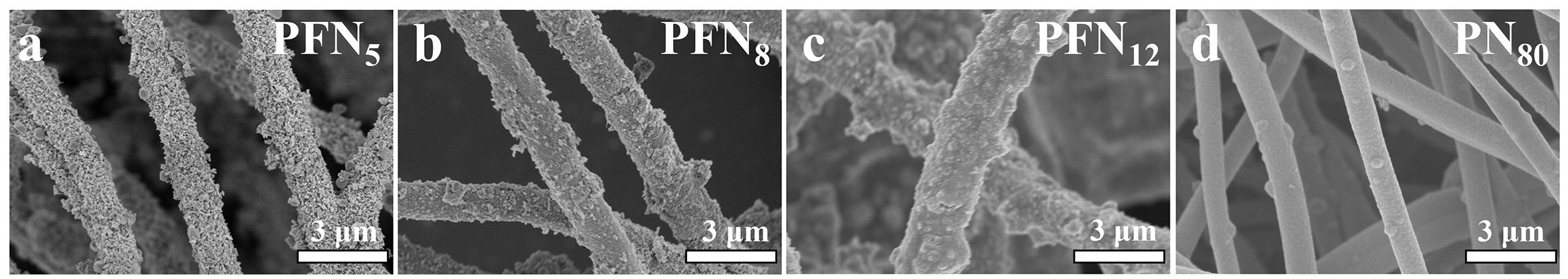


**Fig. S8** SEM images of (**a**) PFN_5_, (**b**) PFN_8_, (**c**) PFN_12_ and (**d**) PN_80_


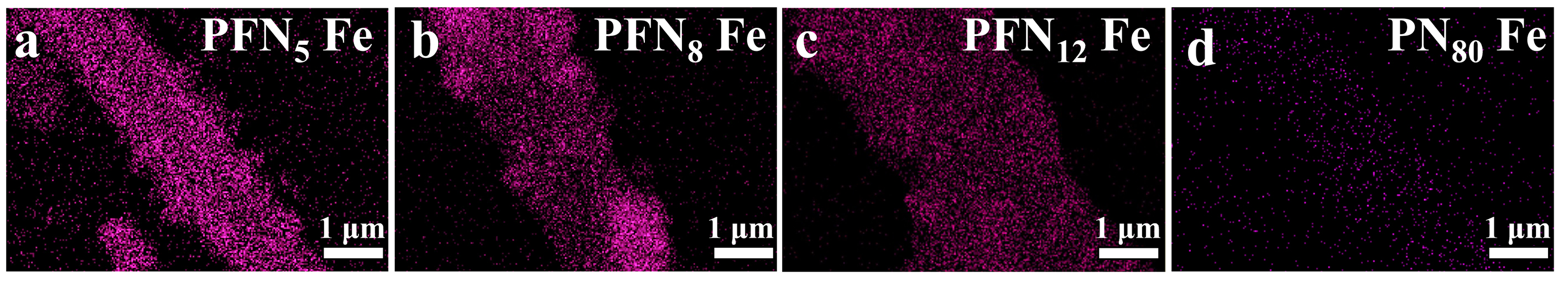


**Fig. S9** EDS mapping images of (**a**) PFN_5_, (**b**) PFN_8_, (**c**) PFN_12_ and (**d**) PN_80_


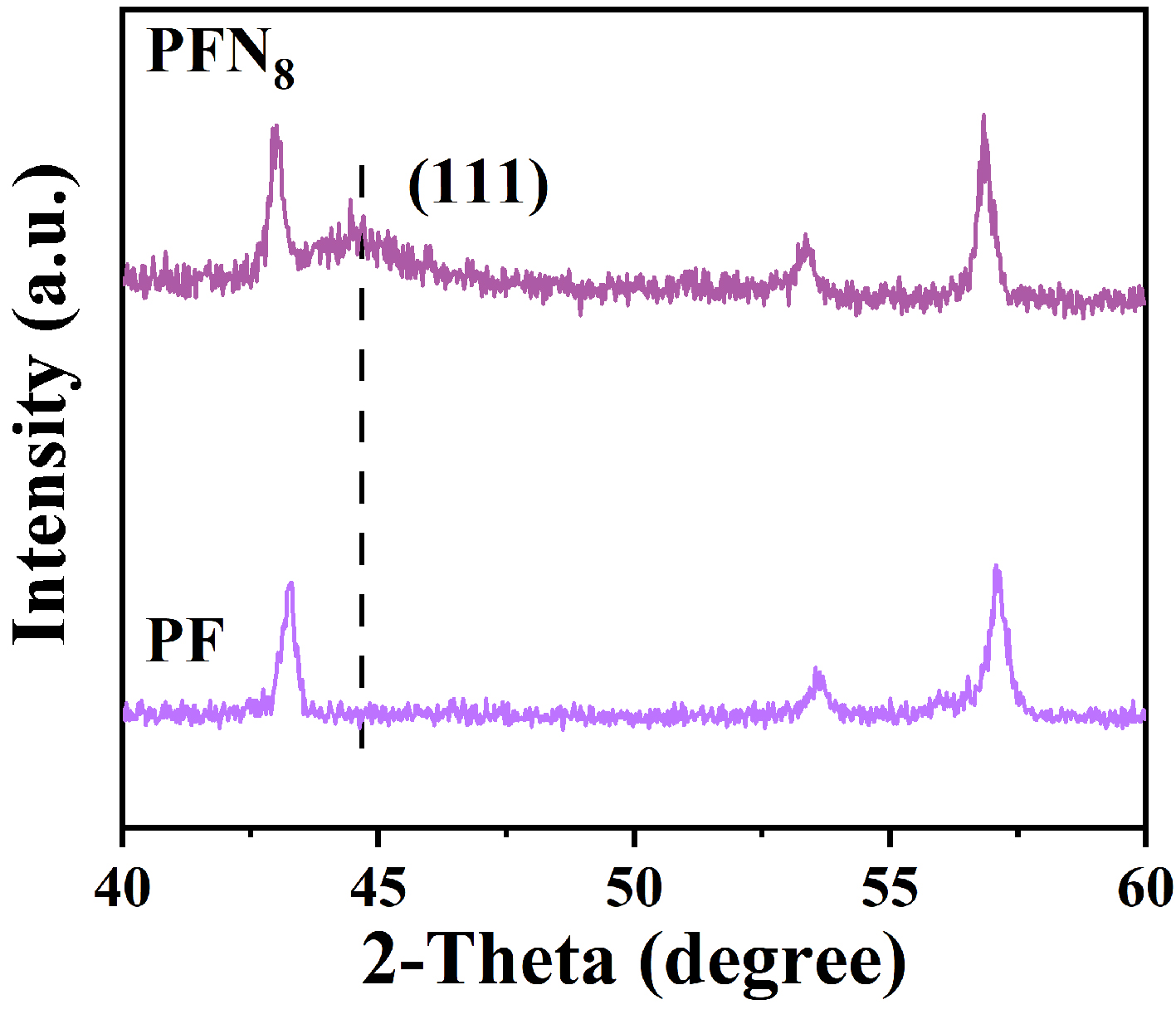


**Fig. S10** XRD patterns of PF and PFN_8_


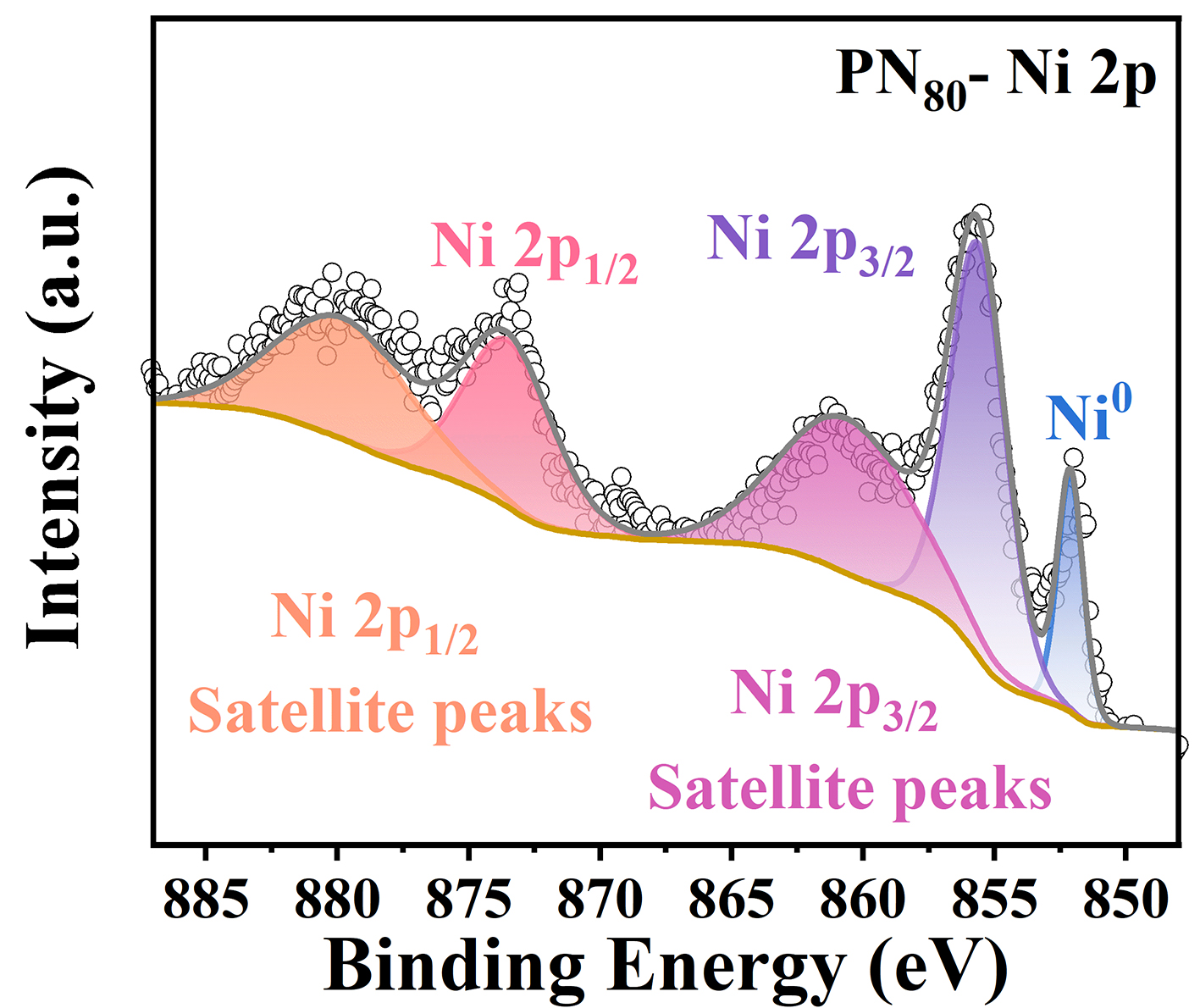


**Fig. S11** High resolution XPS spectra of Ni 2p for PN_80_


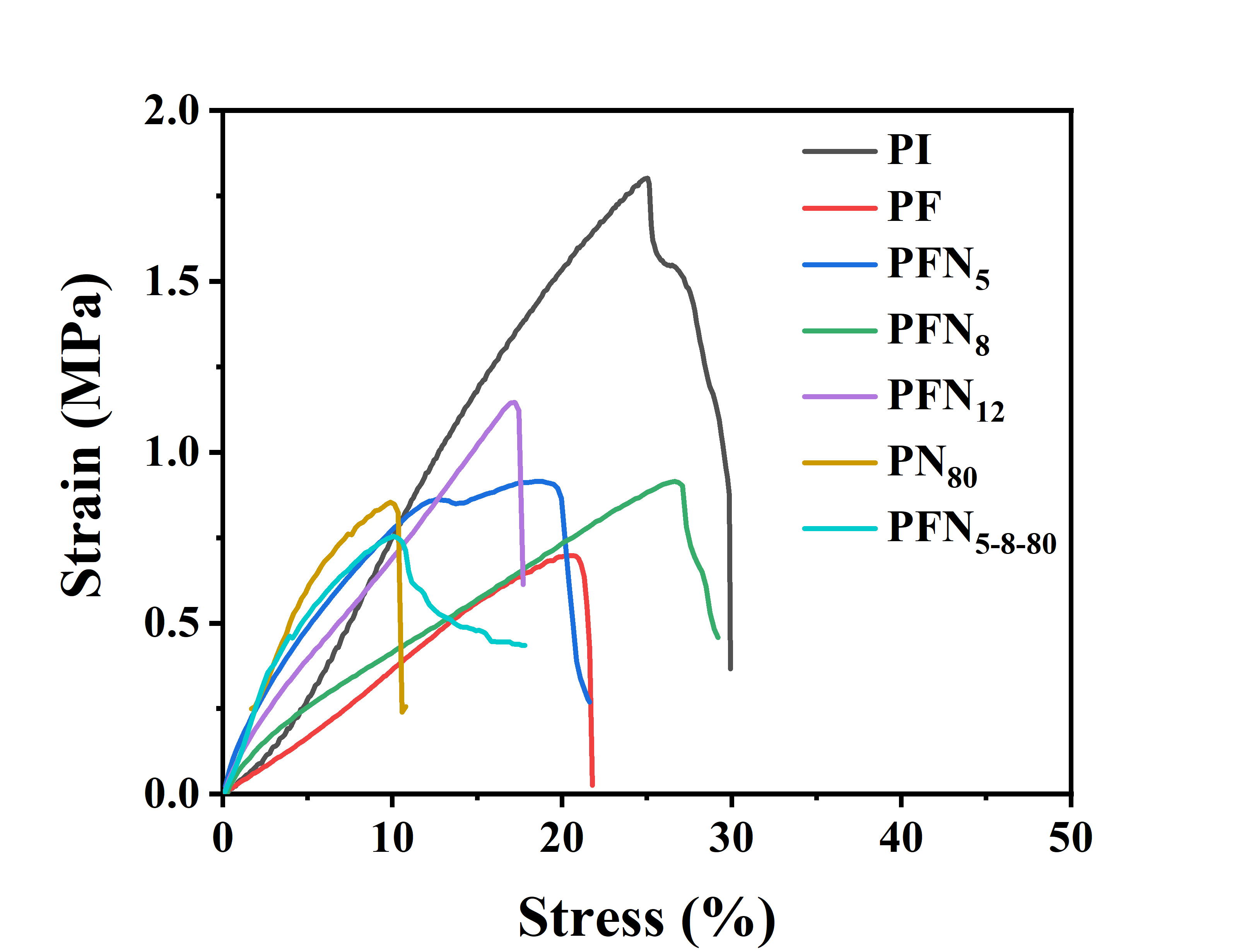


**Fig. S12** Strain-Stress curves of PI, PF, PFN_5_, PFN_8_, PFN_12_, PN_80_, and PFN_5-8-80_


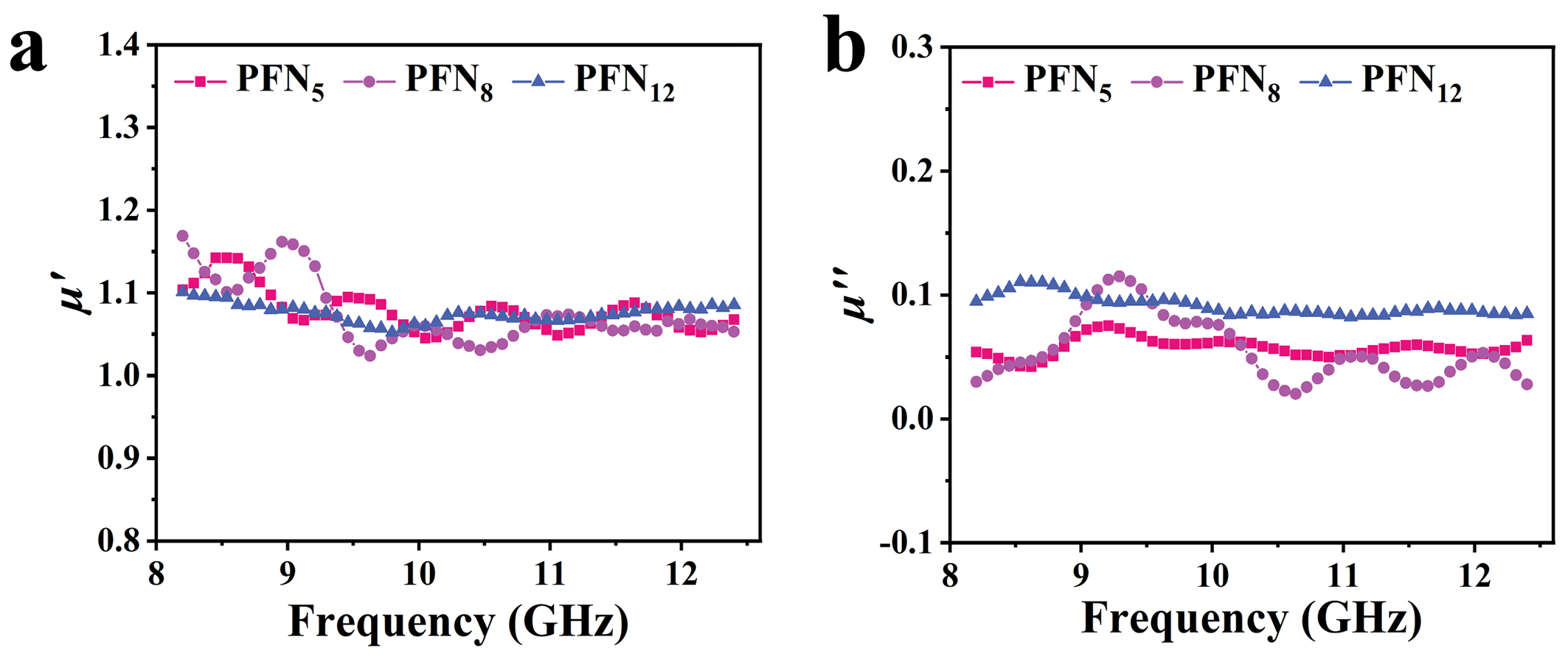


**Fig. S13** (**a**) Real permeability (*μ′*) and (**b**) Imaginary permeability (*μ′′*) of PFN_5_, PFN_8_, and PFN_12_


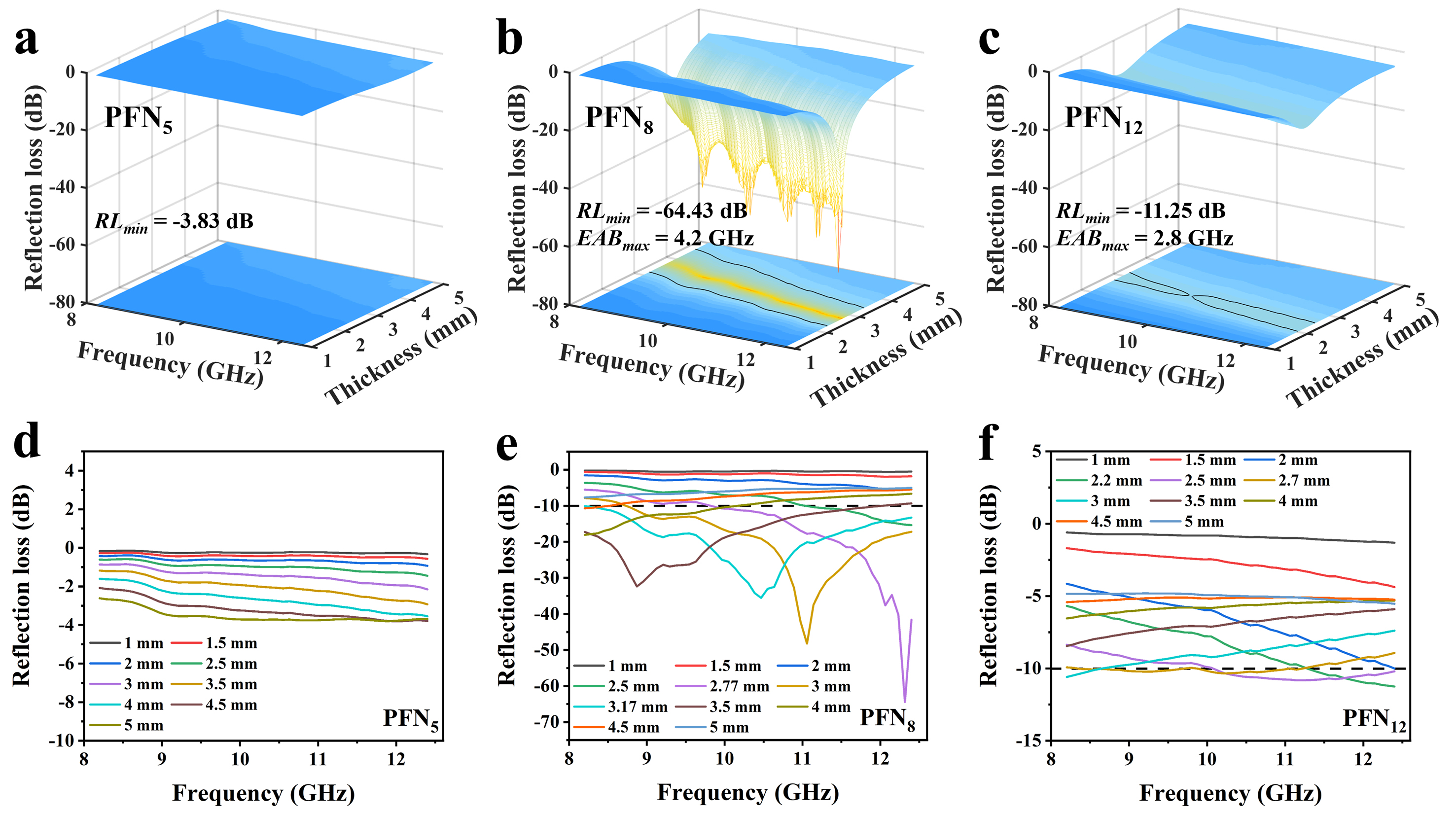


**Fig. S14** 3D reflection loss of (**a**) PFN_5_, (**b**) PFN_8_, and (**c**) PFN_12_. 2D reflection loss of (**d**) PFN_5_, (**e**) PFN_8_, and (**f**) PFN_12_


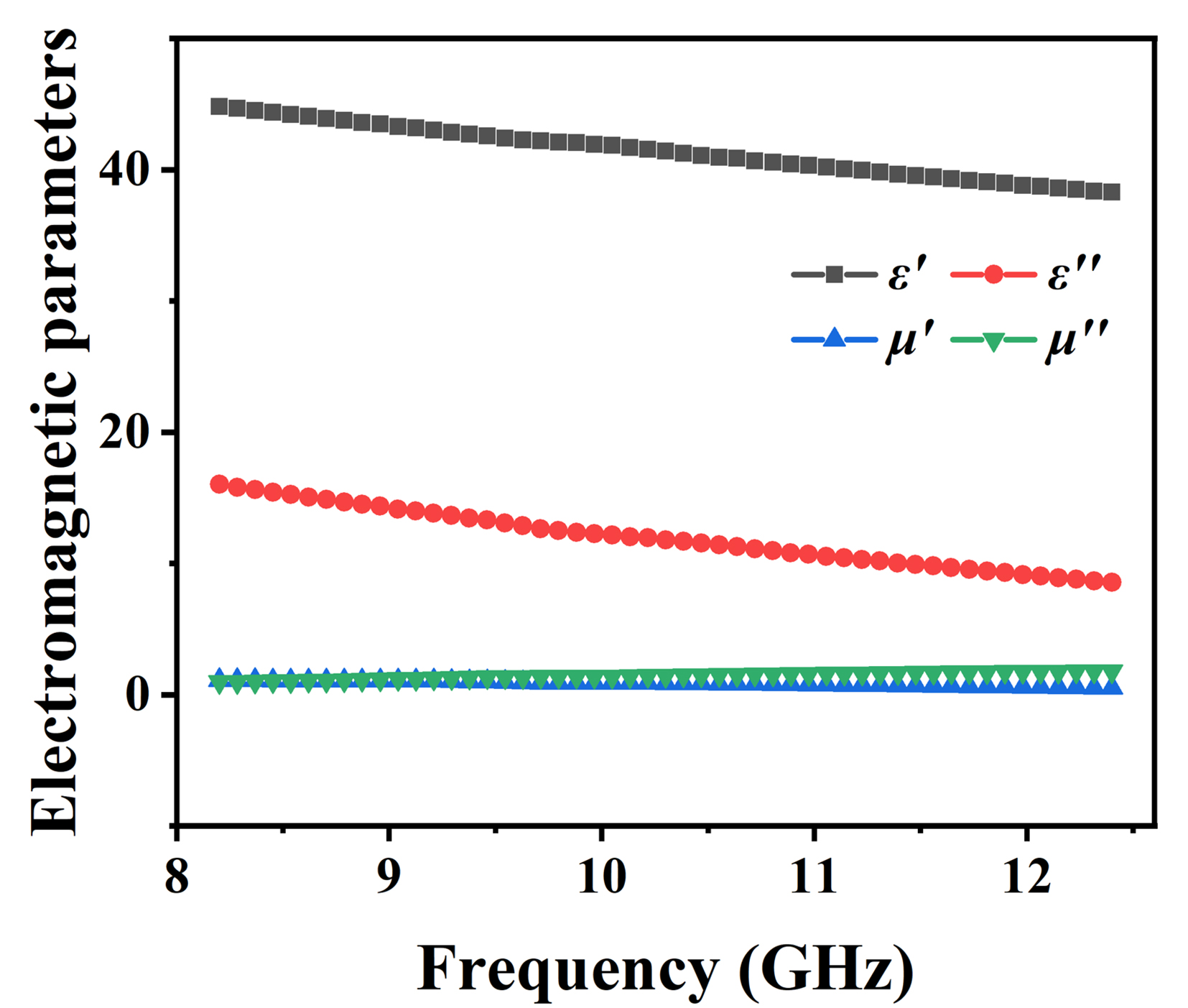


**Fig. S15** Electromagnetic parameters (*ε′*, *ε′′*, *μ′*, and *μ′′*) of PN_80_


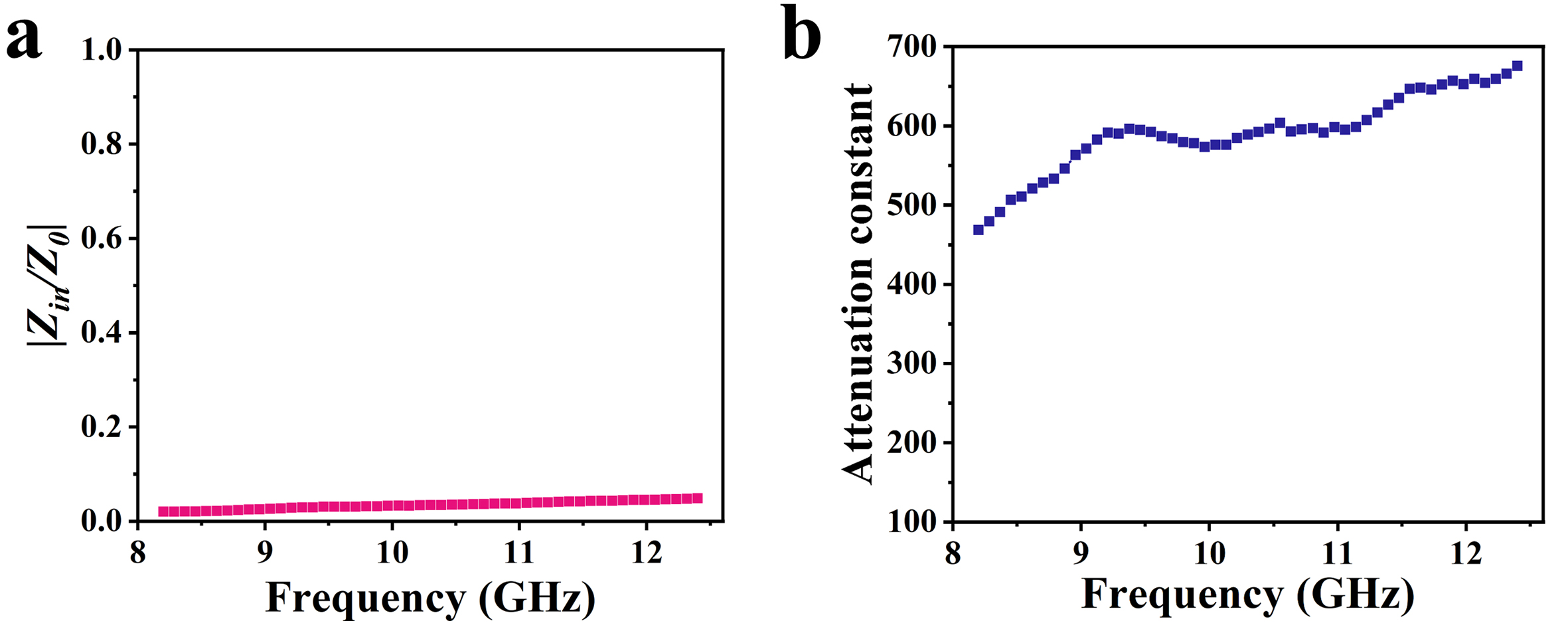


**Fig. S16** (**a**) Impedance matching (|*Z_in_/Z_0_*|) and (**b**) attenuation constant of PN_80_


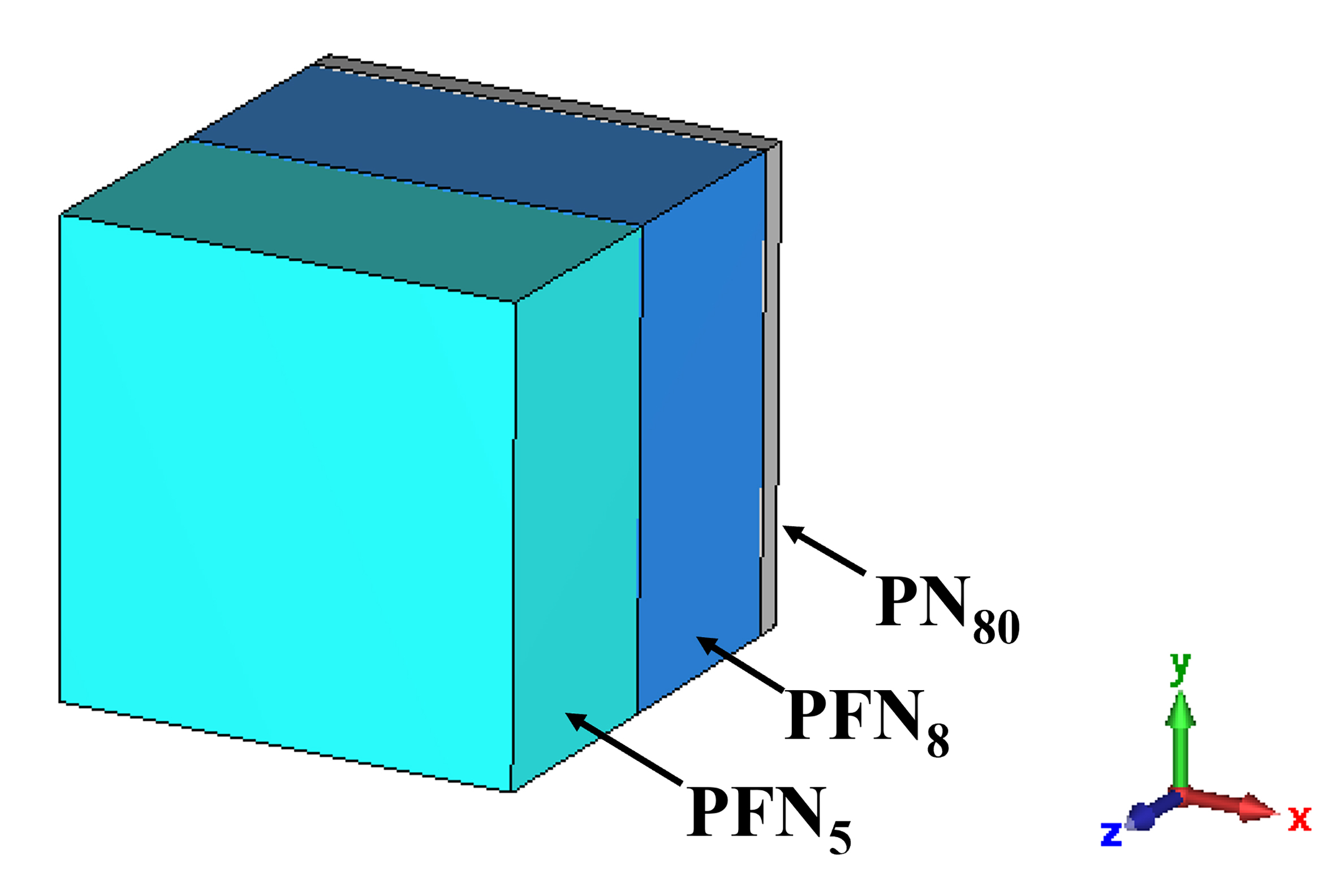


**Fig. S17** Simulation model of PFN_5-8-80_ in CST Studio Suite software


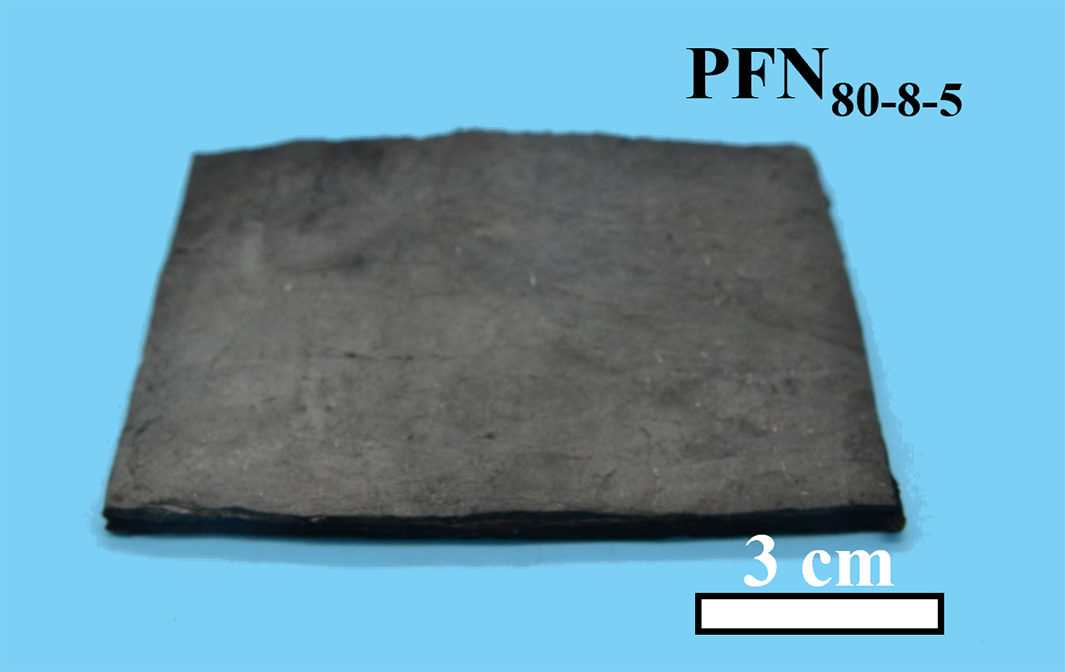


**Fig. S18** Optical photograph of PFN_80-8-5_


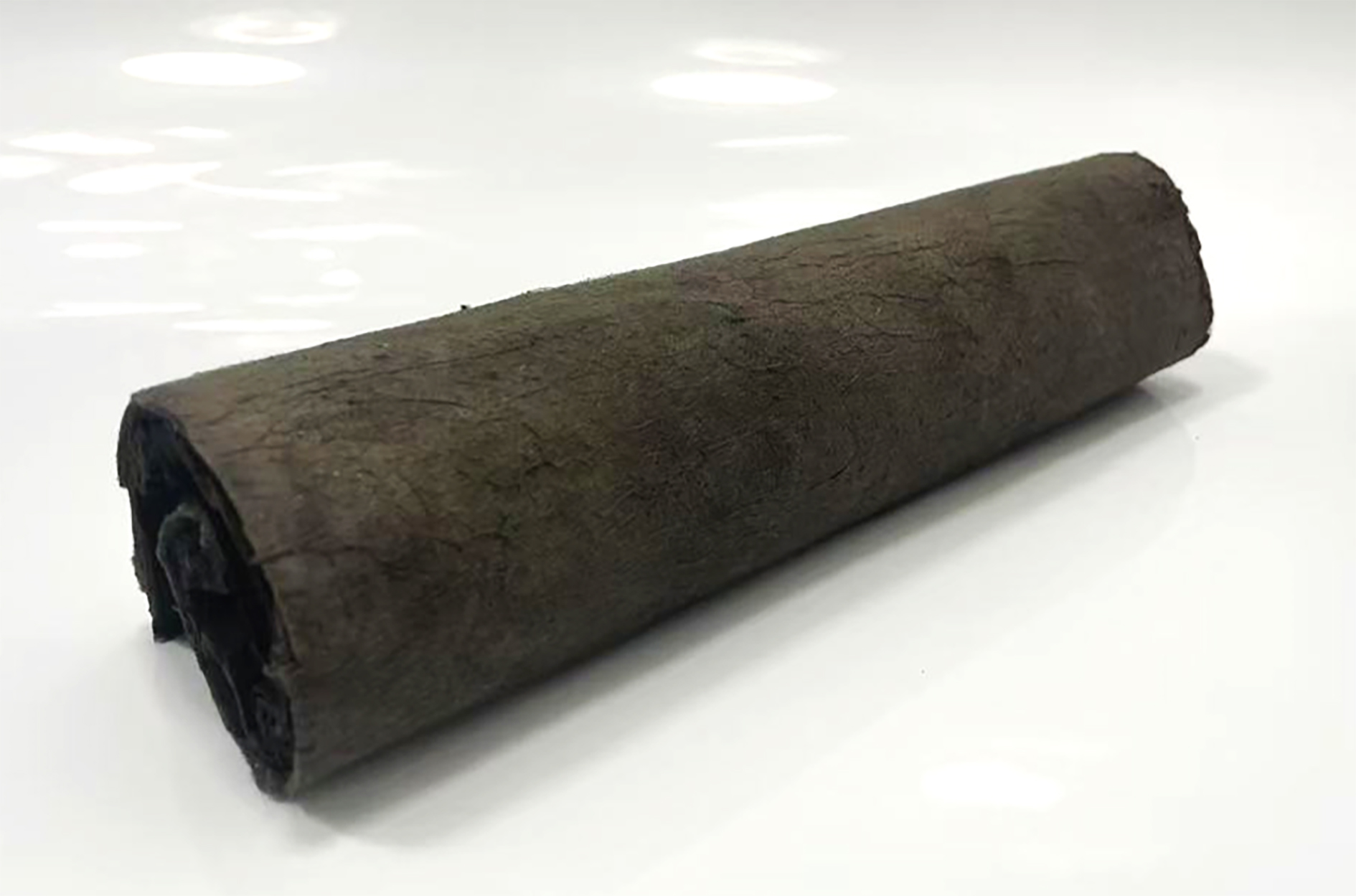


**Fig. S19** Optical photograph of PFN_5-8-80_

**
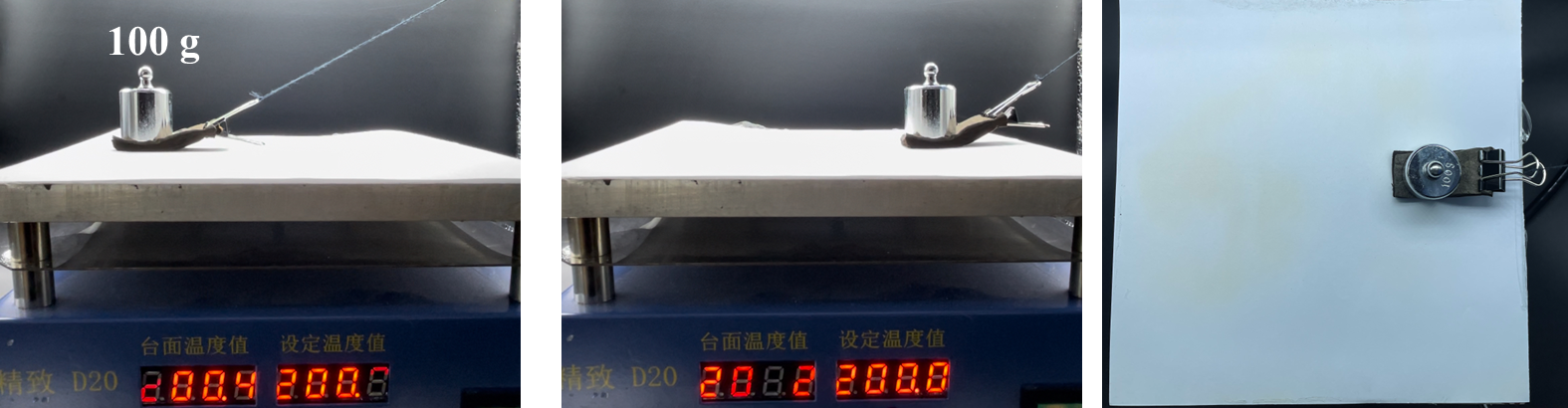
**

**Fig. S20** The bonding force characteristic of PFN_5-8-80_ at 200 °C


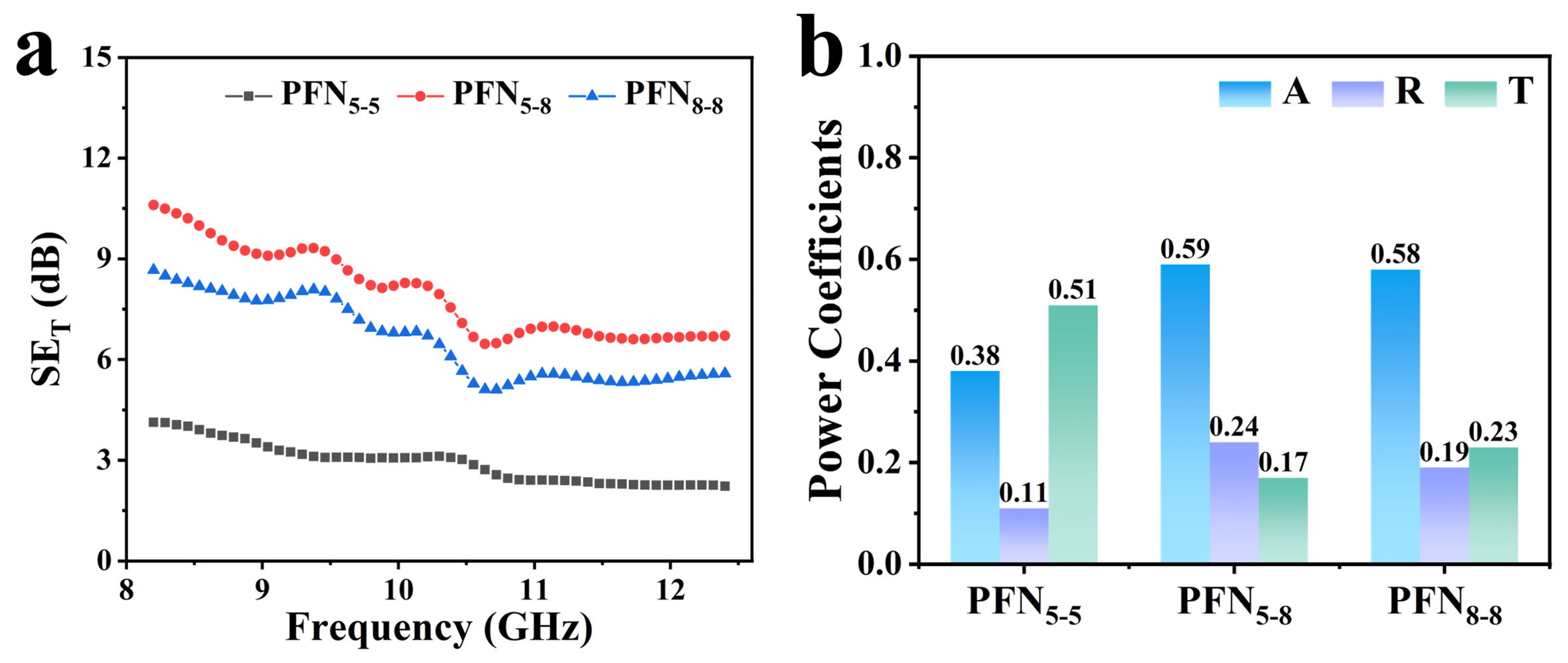


**Fig. S21** (**a**) SE_T_ and (**b**) Power Coefficients of PFN_5-5_, PFN_5-8_, and PFN_8-8_ with a thickness of 5 mm


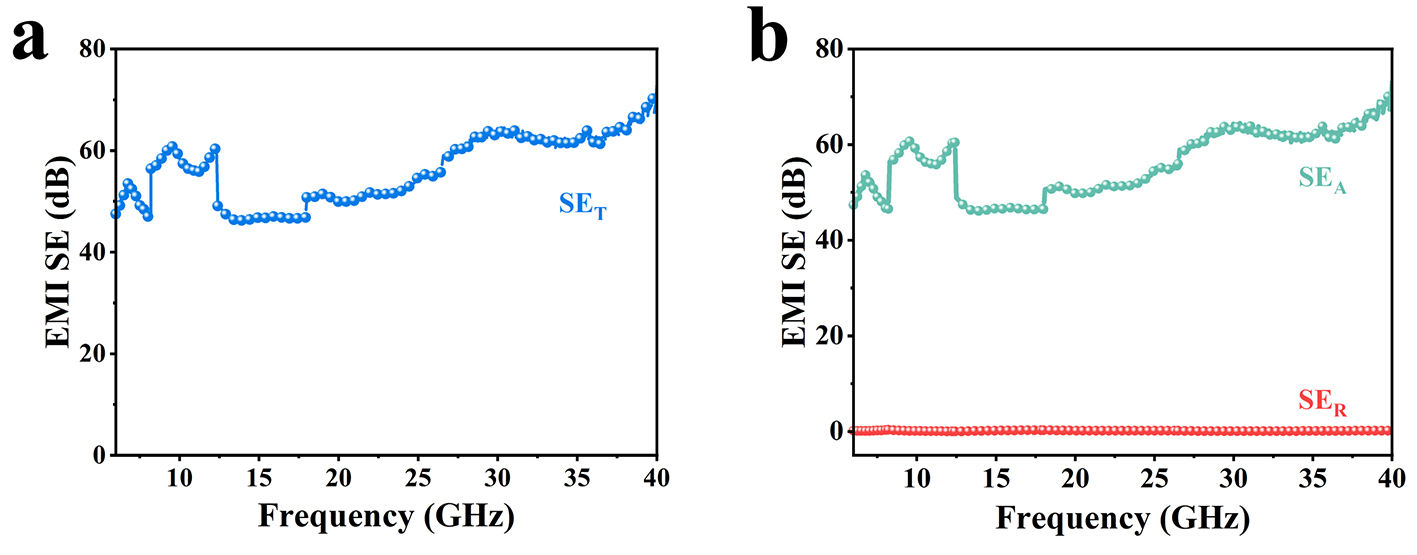


**Fig. S22** (**a**) SE_T_, (**b**) SE_A_ and SE_R_ of PFN_5-8-80_ at 6-40 GHz


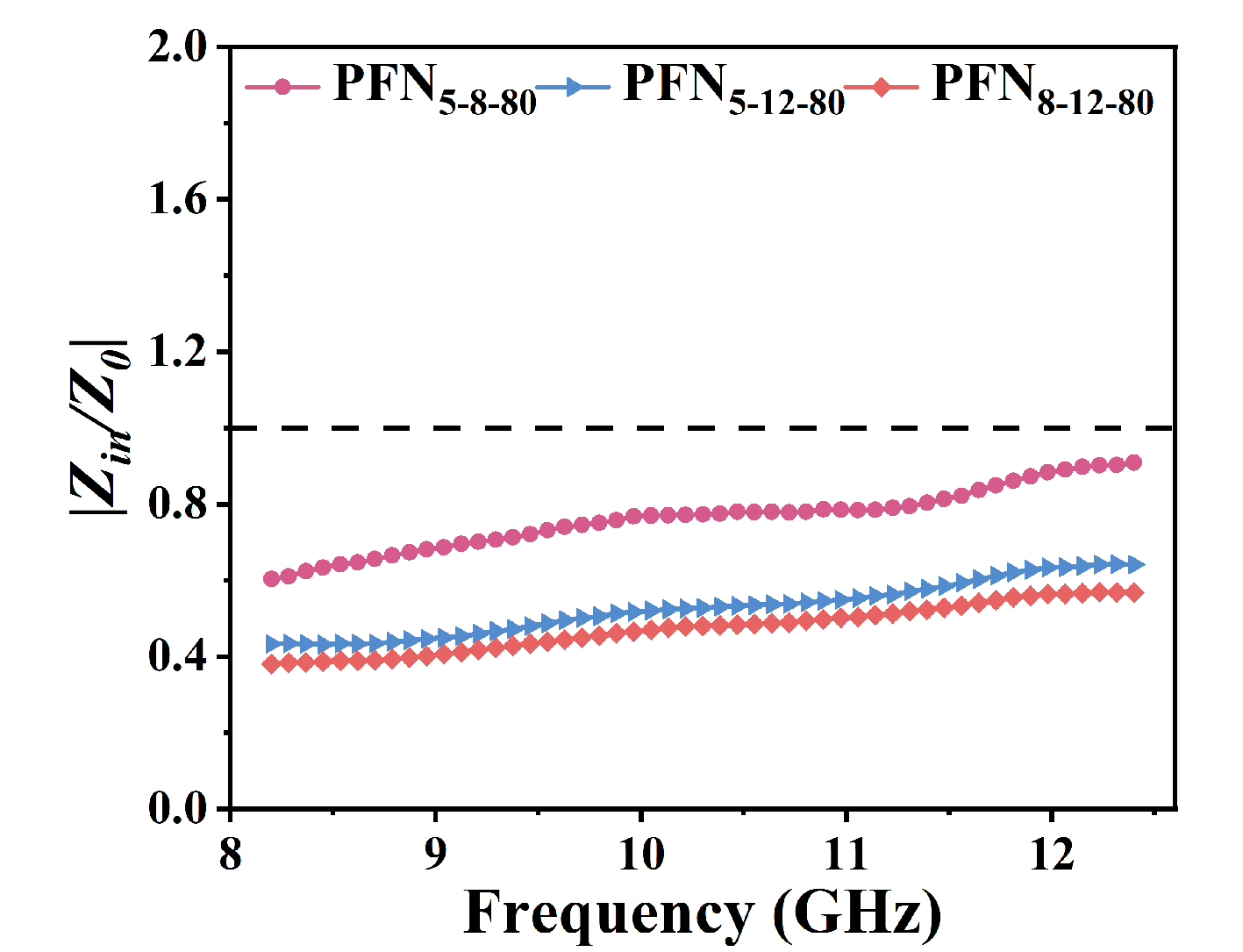


**Fig. S23** Impedance matching (*|Z_in_/Z_0_|*) of PFN_5-8-80_, PFN_5-12-80_, and PFN_8-12-80_


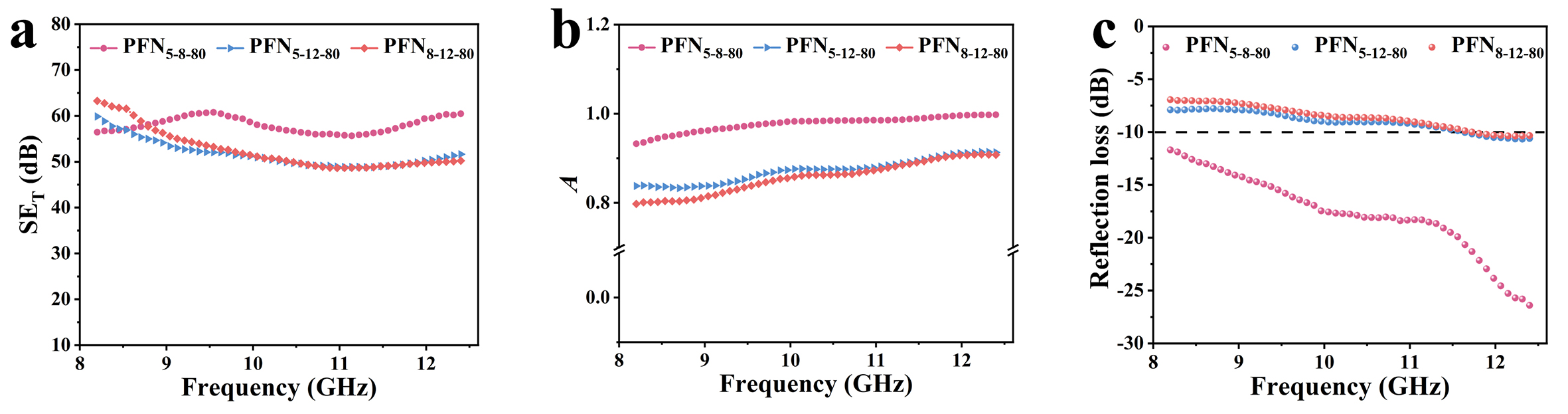


**Fig. S24** (**a**) SE_T_, (**b**) A, and (**c**) Reflection loss of PFN_5-8-80_, PFN_5-12-80_, and PFN_8-12-80_


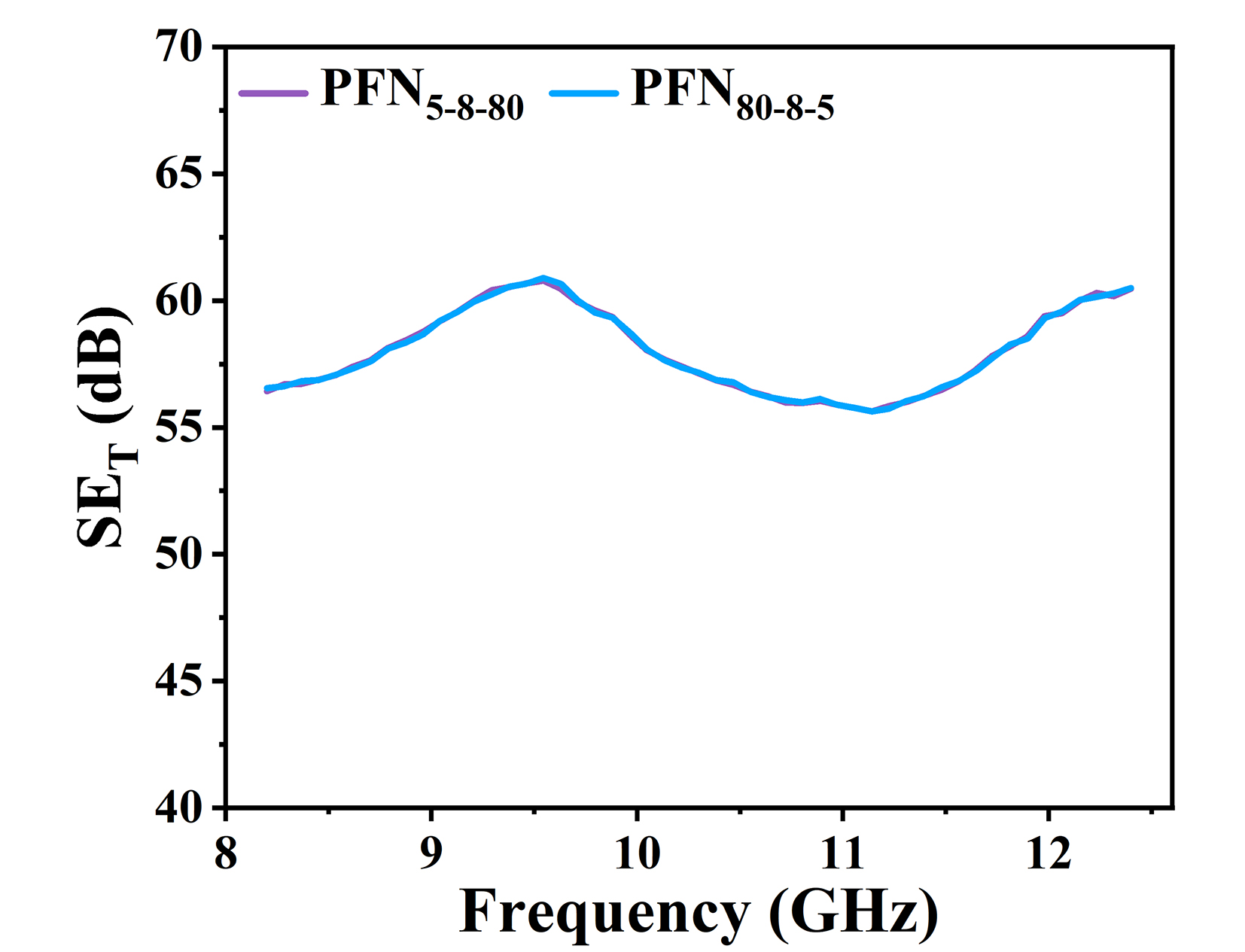


**Fig. S25** SE_T_ of PFN_5-8-80_ and PFN_80-8-5_


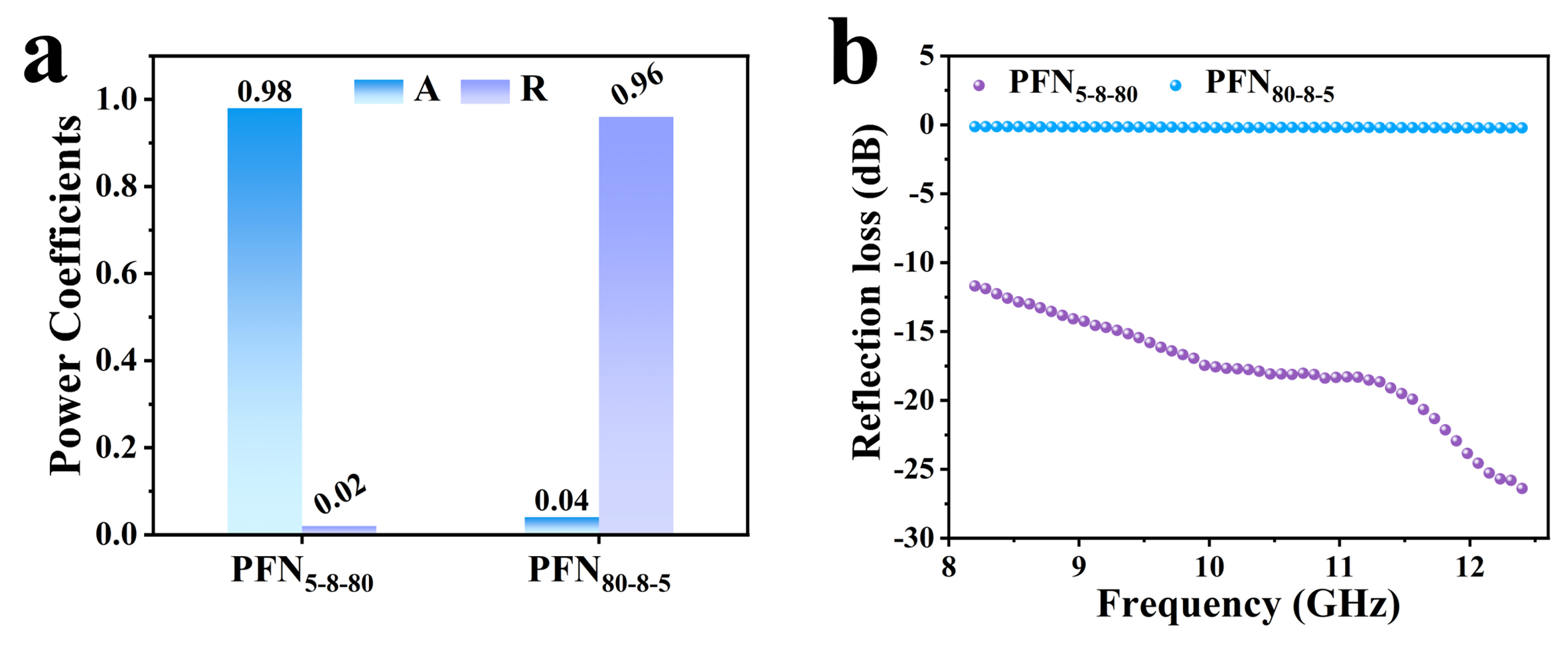


**Fig. S26** (**a**) Power Coefficients and (**b**) Reflectivity of PFN_5-8-80_ and PFN_80-8-5_


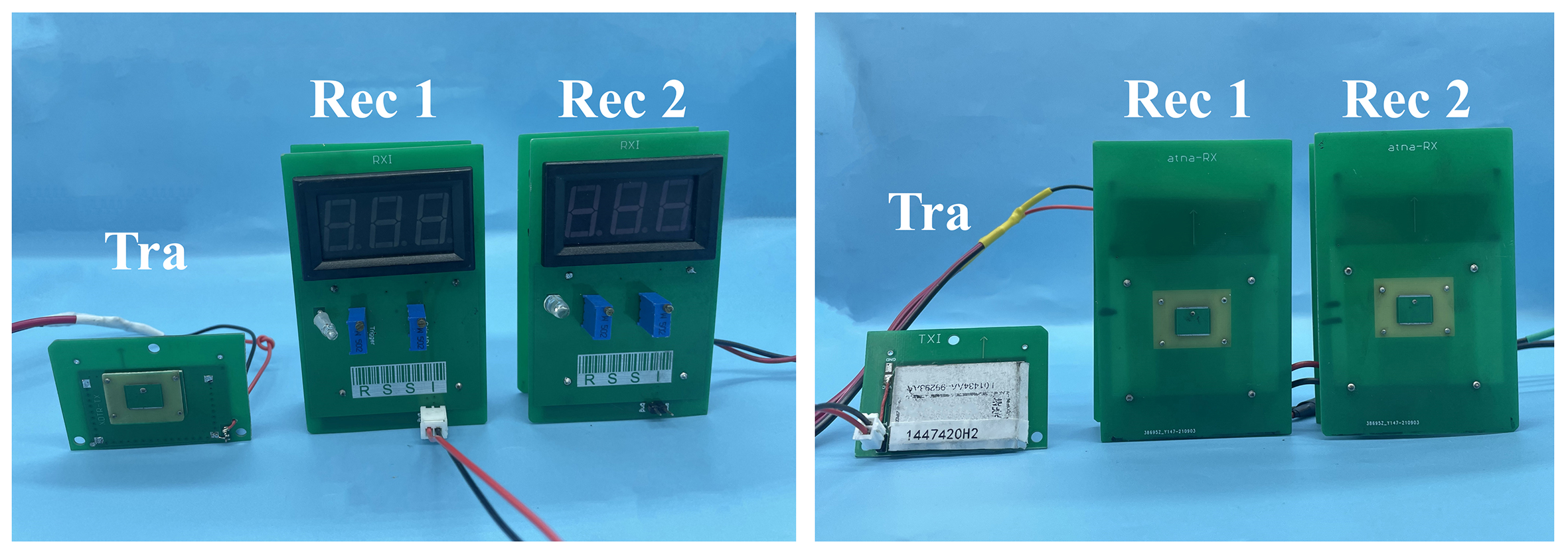


**Fig. S27** Photographs of 10.5 GHz electromagnetic demonstration devices


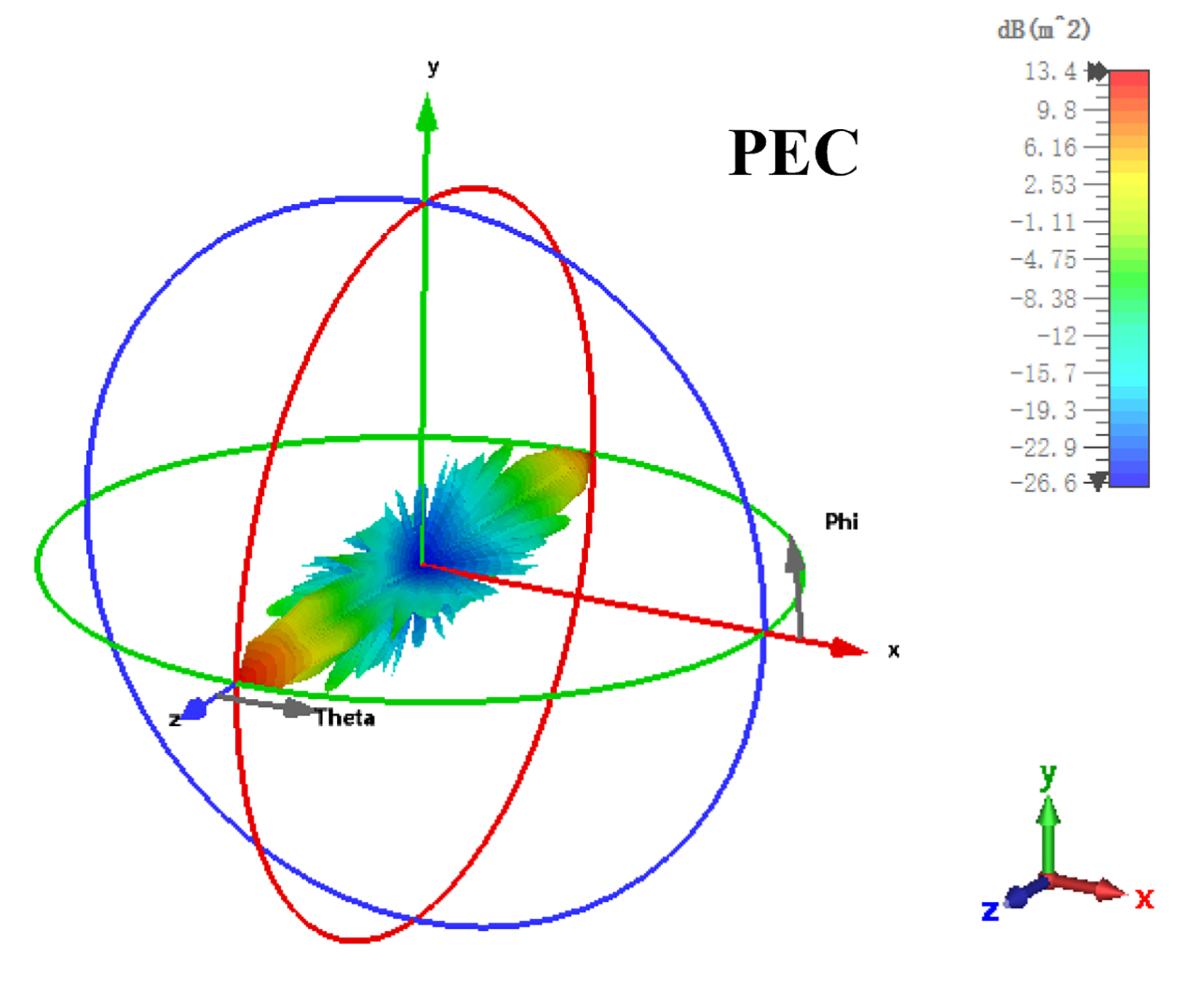


**Fig. S28** 3D RCS of PEC


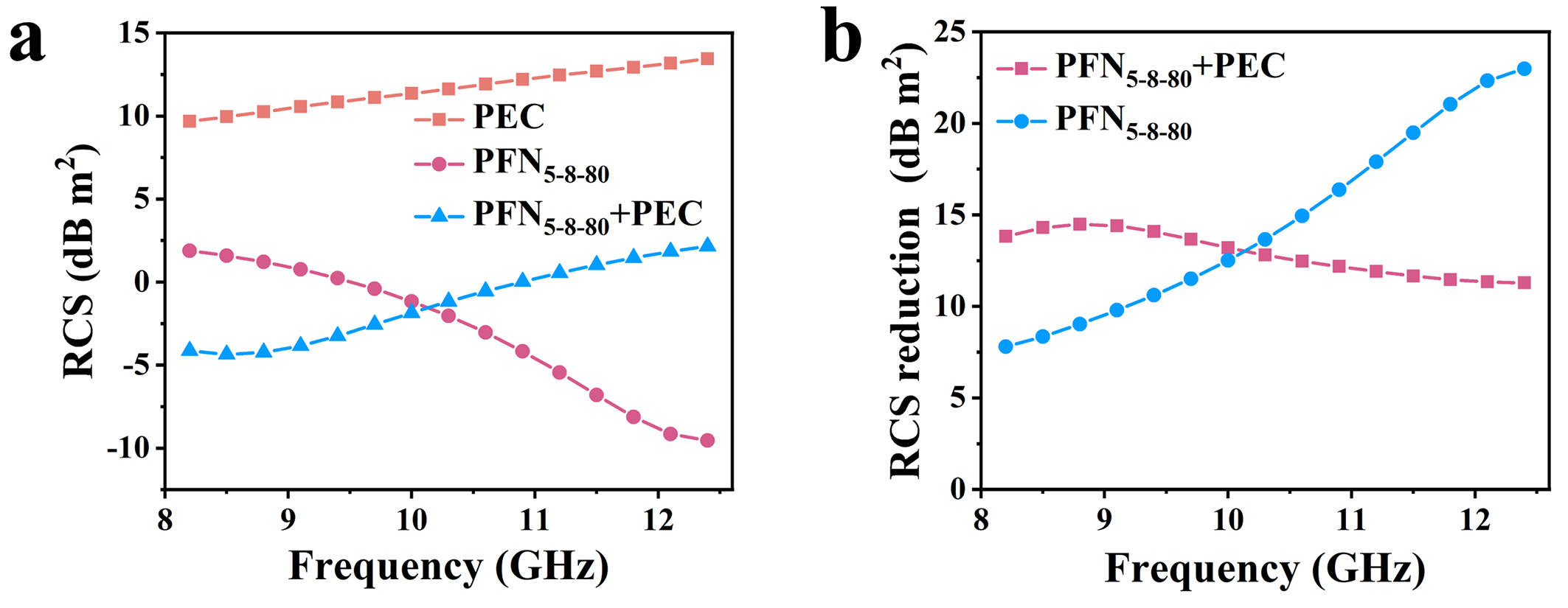


**Fig. S29** (**a**) RCS values of PEC, PFN_5-8-80_ and PFN_5-8-80_+PEC. (**b**) RCS reduction of PFN_5-8-80_ and PFN_5-8-80_+PEC in comparison with PEC

The RCS reduction value under different degree and frequency is caiculated by the following equation:

(1) RCS reduction (PFN_5-8-80_) = RCS (PEC) - RCS (PFN_5-8-80_)

(2) RCS reduction (PFN_5-8-80_+PEC) = RCS (PEC) - RCS (PFN_5-8-80_+PEC)


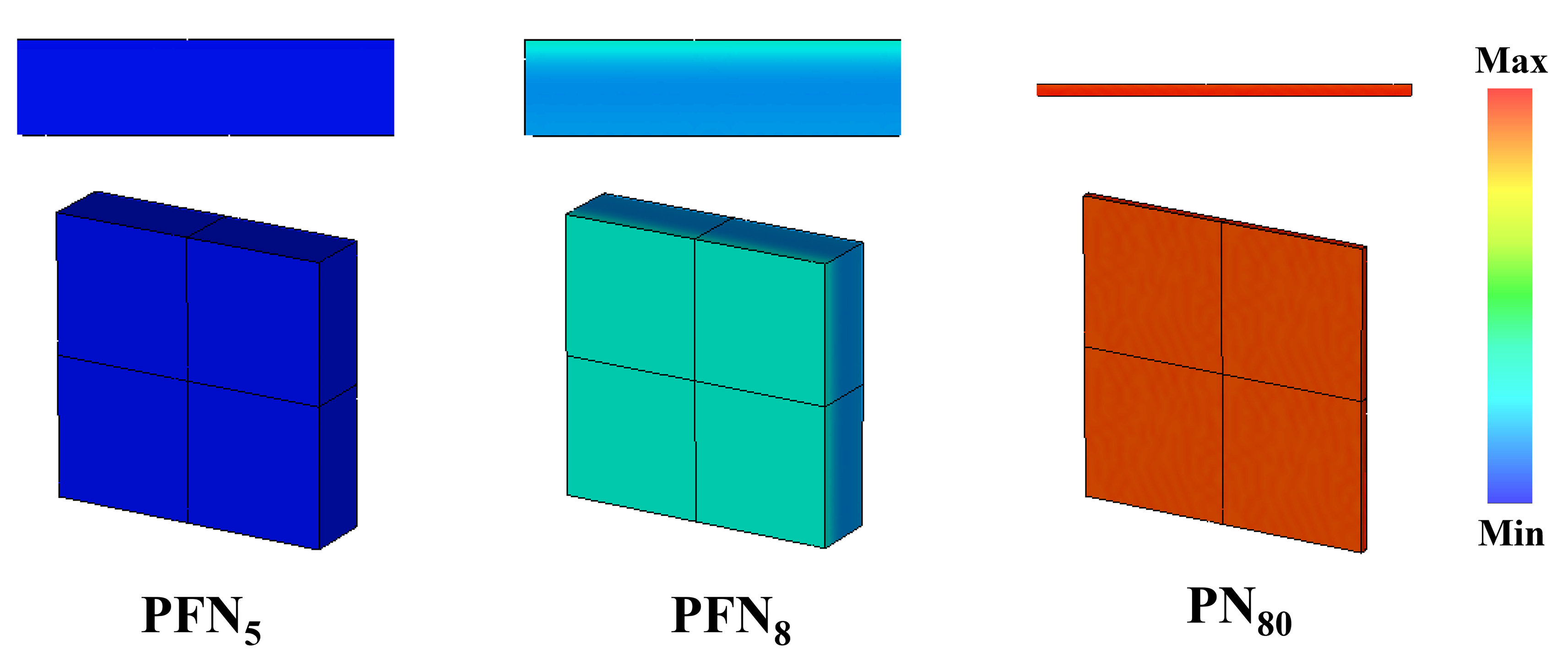


**Fig. S30** Power loss density of PFN_5_, PFN_8_, and PN_80_ at 12 GHz


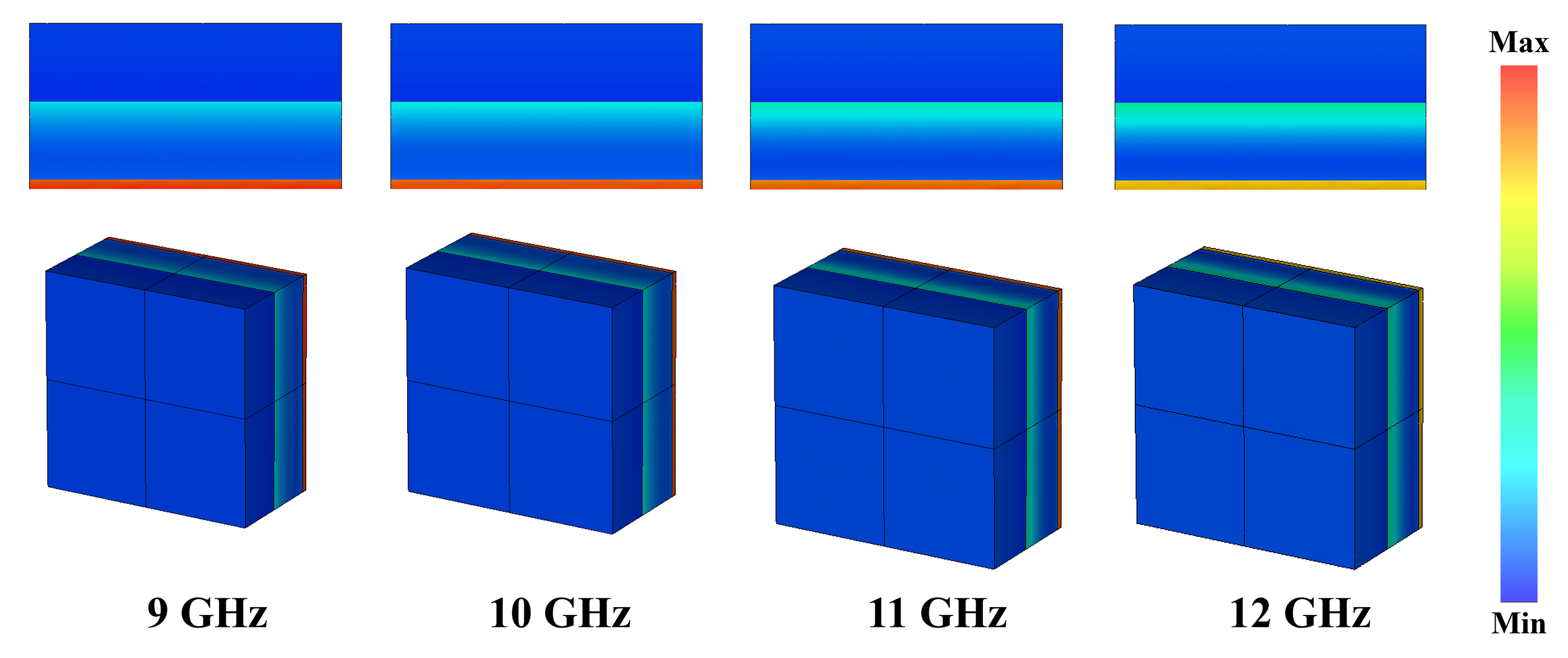


**Fig. S31** Power loss density of PFN_5-8-80_ at 9, 10, 11, and 12 GHz


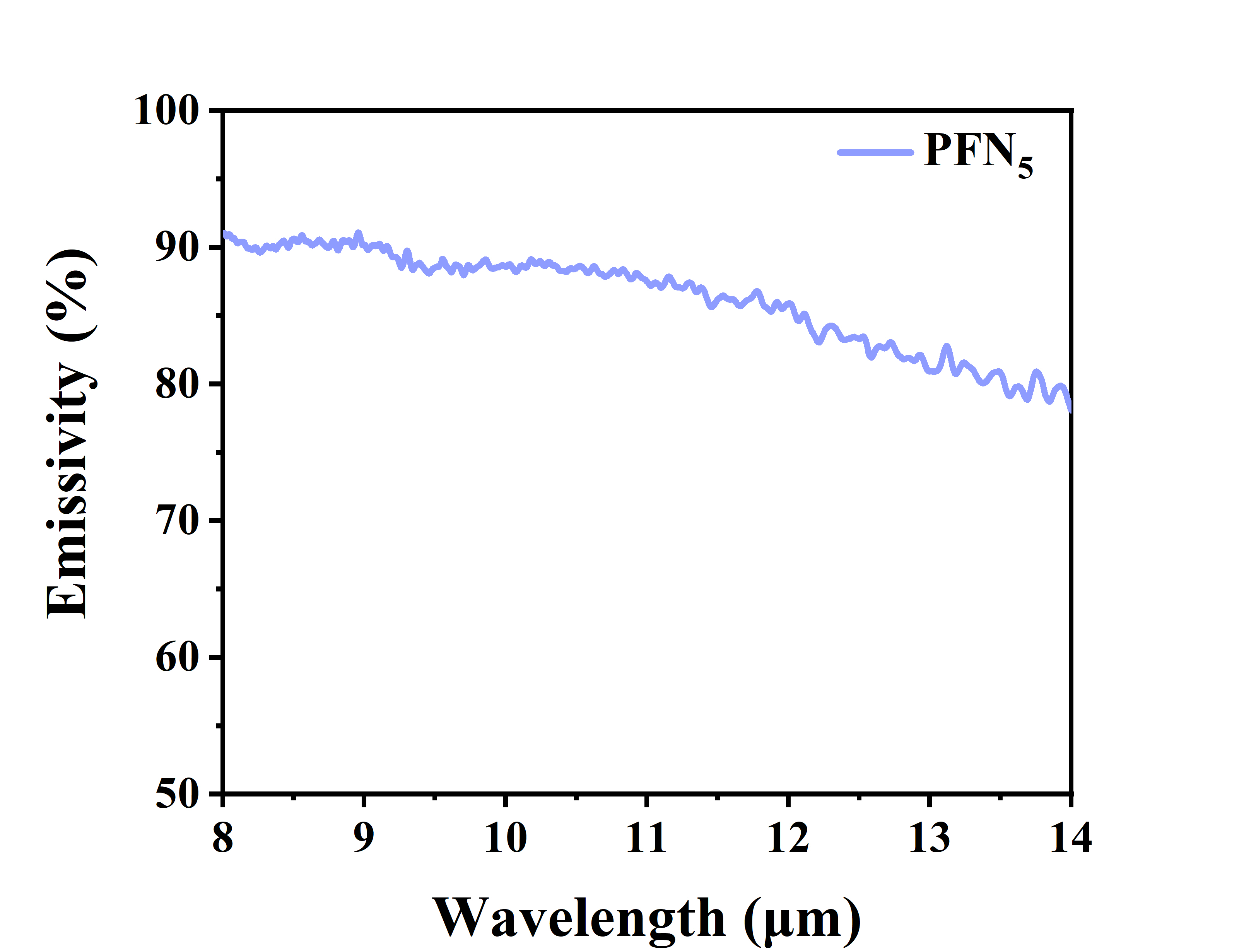


**Fig. S32** The infrared emissivity of PFN_5_


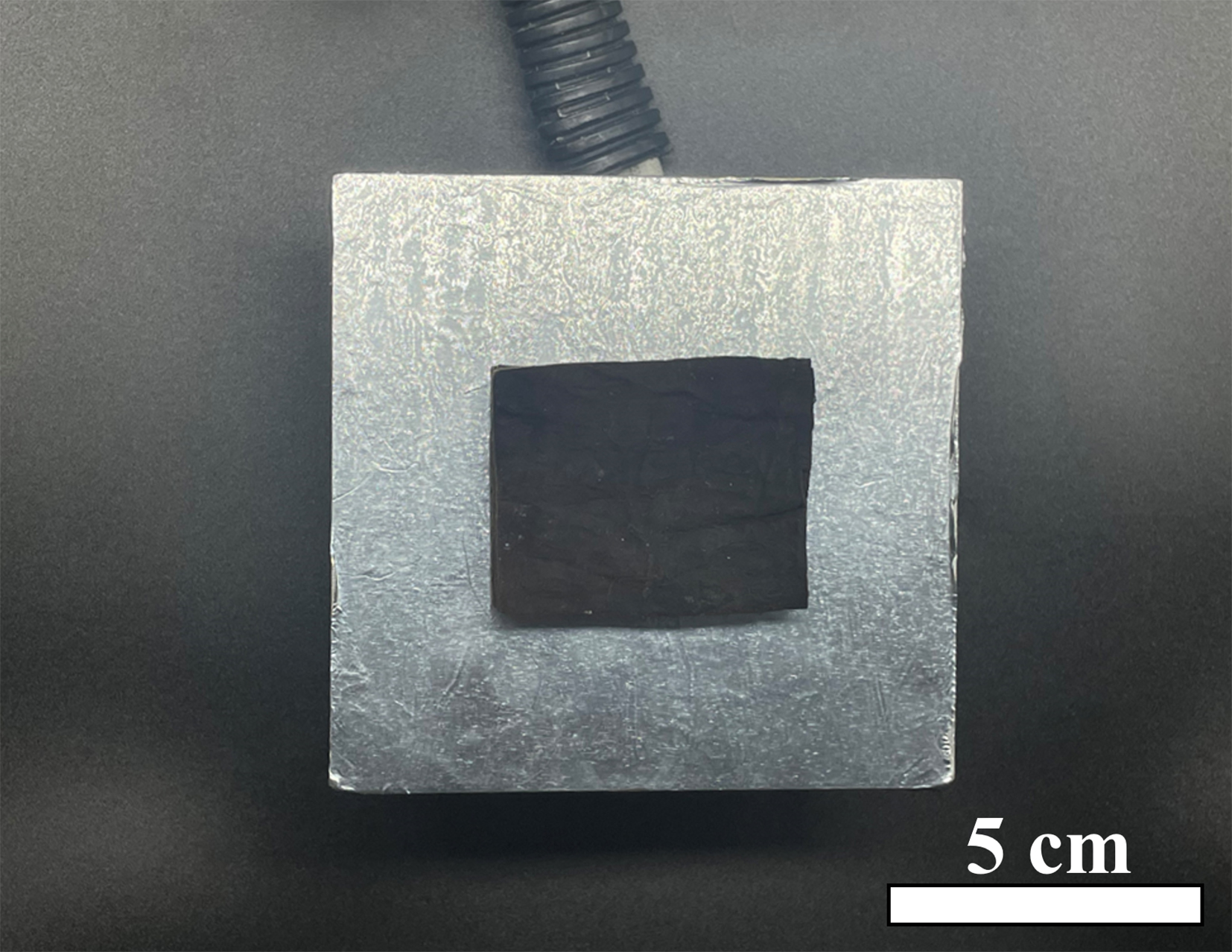


**Fig. S33** Photography of infrared stealth testing for PFN_5-8-80_ on a thermal stage


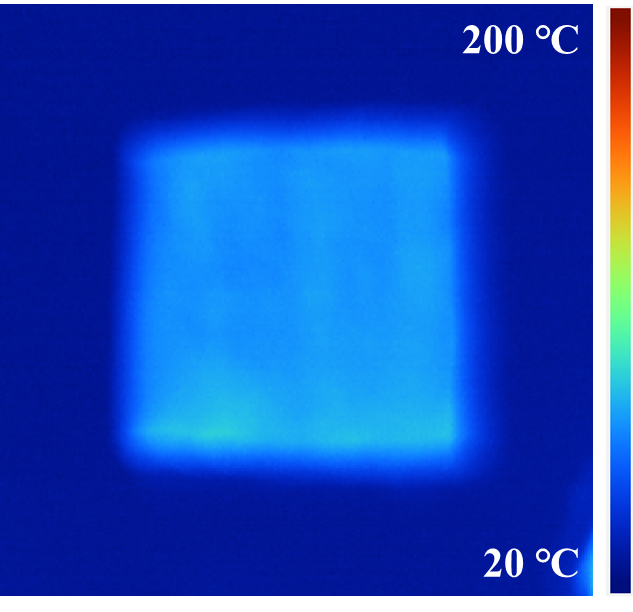


**Fig. S34** Infrared image of PFN_5-8-80_ at a thermal stage of 200 °C for 24 h

**Table S1** Comparison for radar stealh performances of recently reported materials

| **Samples** | **Density**  **(g cm^-3^)** | **Thickness**  **(mm)** | ***RL_min_***  **(dB)** | ***EAB_max_***  **(GHz)** | **Refs.** |
| --- | --- | --- | --- | --- | --- |
| H_f_/epoxy-L(OBN)_f_/epoxy | / | 5.6 | -19.4 | 9.8 | [44] |
| PI/CB | 0.27 | 25 | -44.8 | 16 | [45] |
| PI/GN/Fe_3_O_4_/SiO_2_ | 0.40 | 15 | -25.5 | 22.4 | [46] |
| NiCoFe@C |  | 2.0 | -31 | 11 | [47] |
| CNT@ZIS/CNF | 0.04 | 2.7 | -13 | 3.4 | [48] |
| CB/CIP |  | 8.6 | ~ -32 | 14.3 | [49] |
| ACDMS | / | 25 | -37.9 | 35.9 | [50] |
| SiC-BN@CNT | / | 15 | ~ -37 | 34.62 | [51] |
| 3D resistance film | / | 11 | -15.9 | 22.3 | [52] |
| CuHT-FCIP-EP | 0.89 | 9.3 | -51 | 38 | [53] |
| PFN_5-8-80_ | 0.15 | 5.3 | -23.87 | 22.1 | This work |

**Movie S1** Demonstration of electromagnetic interference shielding and electromagnetic absorption capability of PFN_5-8-80_ at 10.5 GHz.

**Movie S2** Demonstration of electromagnetic interference shielding and electromagnetic reflection capability of PFN_80-8-5_ at 10.5 GHz.

**Movie S3** Demonstration of Real-Time Reflection loss of PFN_5-8-80_ at 8.2 GHz.

**Movie S4** Demonstration of Real-Time Reflection loss of PFN_80-8-5_ at 8.2 GHz.

**Movie S5** Demonstration of Real-Time Reflection loss of PFN_5-8-80_ and PFN_80-8-5_ at 8.2 GHz.
